# Competitive and substrate limited environments drive metabolic heterogeneity for comammox *Nitrospira*

**DOI:** 10.1038/s43705-023-00288-8

**Published:** 2023-08-29

**Authors:** Eloi Martinez-Rabert, Cindy J. Smith, William T. Sloan, Rebeca Gonzalez-Cabaleiro

**Affiliations:** 1 James Watt School of Engineering, Infrastructure and Environment Research Division, University of Glasgow, Advanced Research Centre, Glasgow, UK; 2 Department of Biotechnology, Delft University of Technology, Delft, The Netherlands

**Keywords:** Theoretical ecology, Food webs

## Abstract

*Nitrospira* has been revealed as a high versatile genus. Although previously considered only responsible for the conversion of nitrite to nitrate, now we know that *Nitrospira* can perform complete ammonia oxidation to nitrate too (comammox). Comammox activity was firstly reported as dominant in extremely limited oxygen environments, where anaerobic ammonia oxidation was also occurring (anammox). To explain the comammox selection, we developed an Individual-based Model able to describe *Nitrospira* and anammox growth in suspended flocs assembled in a dynamic nitrogen and oxygen-limiting environment. All known and hypothesized nitrogen transformations of *Nitrospira* were considered: ammonia and nitrite oxidation, comammox, nitrate-reducing ammonia oxidation, and anaerobic nitrite-reducing ammonia oxidation. Through bioenergetics analysis, the growth yield associated to each activity was estimated. The other kinetic parameters necessary to describe growth were calibrated according to the reported literature values. Our modeling results suggest that even extremely low oxygen concentrations (~1.0 µM) allow for a proportional growth of anammox versus *Nitrospira* similar to the one experimentally observed. The strong oxygen limitation was followed by a limitation of ammonia and nitrite, because anammox, without strong competitors, were able to grow faster than *Nitrospira* depleting the environment in nitrogen. These substrate limitations created an extremely competitive environment that proved to be decisive in the community assembly of *Nitrospira* and anammox. Additionally, a diversity of metabolic activities for *Nitrospira* was observed in all tested conditions, which in turn, explained the transient nitrite accumulation observed in aerobic environments with higher ammonia availability.

## Introduction

Nitrification, the biological oxidation of ammonia to nitrate, is a crucial process of the nitrogen cycle in natural and engineered systems. Formerly, nitrification was considered a two-step process where ammonia is first oxidized to nitrite by ammonia-oxidizing microorganisms (bacteria and archaea [1, 2]) and then nitrite is oxidized to nitrate by nitrite-oxidizing bacteria. The understanding of nitrification as a process with an obligated division-of-labor was theoretically questioned [3], and finally refuted by the discovery of nitrite-oxidizing bacteria belonging to the *Nitrospira* genus capable to catalyze both steps of nitrification on their own (complete ammonia oxidation, comammox) [4, 5].

The oligotrophic lifestyle of comammox *Nitrospira* (high affinity for ammonia and high specific growth yield), was already predicted in its theoretical conceptualization [3], and subsequently demonstrated by physiological studies [6, 7]. However, there is still an open debate about the oxygen requirements for the chemoautotrophic growth of comammox *Nitrospira*. Recent studies have found that comammox *Nitrospira* is the dominant ammonia oxidizer in biofilm systems with localized oxygen limitation [8–10], although others have shown that comammox *Nitrospira* require an adequate oxygen supply to growth [11, 12].

One of the first enrichments of comammox *Nitrospira* was obtained together with anammox bacteria without any oxygen supply (oxygen was not detected by an oxygen sensor with a detection limit of 3.1 µM of O_2_) [5]. Given this apparent capacity of comammox *Nitrospira* to survive under so limited oxygen conditions, Kleerebezem and Lücker (2021) [13] proposed two novel cyclic ammonia oxidation processes: (*i*) ammonia oxidation with combined use of oxygen and nitrate as electron acceptors (nitrate-reducing ammonia oxidation), and (*ii*) ammonia oxidation with nitrite as electron acceptor via intracellular production of molecular oxygen (anaerobic nitrite-reducing ammonia oxidation).

Although the available energy from the oxidation of nitrite is limited, the members of the *Nitrospira* genus are ubiquitous in natural and engineered systems [14]. This survival capacity is explained by its physiological versatility and metabolic flexibility (i.e., their ability to obtain energy using alternative electron donor and electron acceptor such as formate and nitrate, respectively) [14]. Microbial populations commonly display heterogenous gene expression profiles that result in metabolic differences between individuals of the same population (referred as metabolic or phenotypic heterogeneity) [15–17]. This diversity can promote the persistence of a microbial population in fluctuating environments and also enable the collaboration between individuals of the same population (e.g., intra-specific commensalism [18], syntrophism [19] or protection [20]) increasing their ability to survive under nutrient-limiting conditions.

For comammox *Nitrospira*, currently reported catabolic activities include ammonia oxidation (AO), nitrite oxidation (NO) and complete ammonia oxidation (CMX) (Eqs. (1)–(3)). Their capacity to catalyze nitrate-reducing ammonia oxidation (NRMX) and anaerobic nitrite-reducing ammonia oxidation (An-NRMX) have also been hypothesized (Eqs. (4) and (5)).

1 $${{{{{{{\mathrm{NH}}}}}}}}_4^ + + 1.5{{{{{{{\mathrm{O}}}}}}}}_2 \to {{{{{{{\mathrm{NO}}}}}}}}_2^ - + {{{{{{{\mathrm{H}}}}}}}}_2{{{{{{{\mathrm{O}}}}}}}} + 2{{{{{{{\mathrm{H}}}}}}}}^ + \left( {\Delta {{{{{{{\mathrm{G}}}}}}}}^{{{{{{{{\mathrm{o}}}}}}}}^\prime } = - 274.7{{{{{{{\mathrm{kJ}}}}}}}}\,{{{{{{{\mathrm{mol}}}}}}}}^{ - 1}} \right)$$

2 $${{{{{{{\mathrm{NO}}}}}}}}_2^ - + 0.5{{{{{{{\mathrm{O}}}}}}}}_2 \to {{{{{{{\mathrm{NO}}}}}}}}_3^ - \left( {\Delta {{{{{{{\mathrm{G}}}}}}}}^{{{{{{{{\mathrm{o}}}}}}}}^\prime } = - 74.1\,{{{{{{{\mathrm{kJ}}}}}}}}\,{{{{{{{\mathrm{mol}}}}}}}}^{ - 1}} \right)$$

3 $${{{{{{{\mathrm{NH}}}}}}}}_4^ + + 2{{{{{{{\mathrm{O}}}}}}}}_2 \to {{{{{{{\mathrm{NO}}}}}}}}_3^ - + {{{{{{{\mathrm{H}}}}}}}}_2{{{{{{{\mathrm{O}}}}}}}} + 2{{{{{{{\mathrm{H}}}}}}}}^ + \left( {\Delta {{{{{{{\mathrm{G}}}}}}}}^{{{{{{{{\mathrm{o}}}}}}}}^\prime } = - 348.9\,{{{{{{{\mathrm{kJ}}}}}}}}\,{{{{{{{\mathrm{mol}}}}}}}}^{ - 1}} \right)$$

4 $${{{{{{{\mathrm{NH}}}}}}}}_4^ + + {{{{{{{\mathrm{O}}}}}}}}_2 + {{{{{{{\mathrm{NO}}}}}}}}_3^ - \to 2{{{{{{{\mathrm{NO}}}}}}}}_2^ - + {{{{{{{\mathrm{H}}}}}}}}_2{{{{{{{\mathrm{O}}}}}}}} + 2{{{{{{{\mathrm{H}}}}}}}}^ + \left( {\Delta {{{{{{{\mathrm{G}}}}}}}}^{{{{{{{{\mathrm{o}}}}}}}}^\prime } = - 200.6\,{{{{{{{\mathrm{kJ}}}}}}}}\,{{{{{{{\mathrm{mol}}}}}}}}^{ - 1}} \right)$$

5 $${{{{{{{\mathrm{NH}}}}}}}}_4^ + + {{{{{{{\mathrm{NO}}}}}}}}_2^ - \to {{{{{{{\mathrm{N}}}}}}}}_2 + 2{{{{{{{\mathrm{H}}}}}}}}_2{{{{{{{\mathrm{O}}}}}}}}\left( {\Delta {{{{{{{\mathrm{G}}}}}}}}^{{{{{{{{\mathrm{o}}}}}}}}^\prime } = - 474.4\,{{{{{{{\mathrm{kJ}}}}}}}}\,{{{{{{{\mathrm{mol}}}}}}}}^{ - 1}} \right)$$

In this study, using an in-silico approach, we have investigated the resilience of comammox *Nitrospira* under different nitrogen and oxygen limited environments (based on early studies [4, 5]), considering its potential metabolic heterogeneity. A population of comammox *Nitrospira* growing together with anammox bacteria under specific environmental conditions, was simulated using an Individual-based Model framework. With this framework, we are able to predict the stable activities of comammox *Nitrospira* that are selected under the different oligotrophic environments tested (predicting its potential metabolic niches). These results are compared with reported experimental observations [4, 5, 11]. We have also evaluated the metabolic activities proposed by Kleerebezem and Lücker (2021) [13] for which no experimental observation has been reported yet.

## Methods

### Growth yield estimation of comammox *Nitrospira*

Growth yield for any metabolic activity can be predicted by evaluating the energy harvested per mole of substrate consumed [21]. Because actual growth yields of hypothetical metabolisms of comammox *Nitrospira* (NRMX and An-NRMX) are unknown, the theoretical value was estimated according to a bioenergetics analysis. To keep the analysis consistent, the methodology was also applied for the growth yield estimation of AO, NO and CMX activities and these values were validated with experimental data (Table 1). Only the growth yield value of anammox bacteria was taken as an average from bibliographic values (0.0515 C-mol biomass per mole of N-NH_3_ [22–25] because the biochemistry of their catabolism is not fully elucidated yet [26, 27].

**Table 1:** Comparison between experimental and estimated growth yield values (in mol_cX_/mol_N_) of comammox *Nitrospira*’s activities with current methodology in this study and TEEM2.

	This study				TEEM2 (∈ = 0.258)				Literature
*x100*	Y_X/NH3_	Y_X/NO2_	Y_X/NO3_	Y_X/O2_	Y_X/NH3_	Y_X/NO2_	Y_X/NO3_	Y_X/O2_	Y_X/eD_^a^
AO	4.09	–	–	2.72	4.13	–	–	2.75	3.91 – 4.27
NO	–	2.42	–	4.84	–	2.61	–	5.23	1.07 – 3.58
CMX	6.51	–	–	3.25	6.00	–	–	3.00	7.53
NRMX	1.67	–	1.67	1.67	2.24	–	2.24	2.24	–
An-NRMX	1.50	1.50	–	–	0.35	0.35	–	–	–
AMX	5.15	5.15	–	–	–	–	–	–	3.40 – 6.60

The growth yield of anammox bacteria (AMX) is also included in the table (average of experimental data from literature).

^a^*eD* electron donor, Y_X/NH3_ for AO, CMX and AMX, Y_X/NO2_ for NO. References: [43, 44] for AO, [45–48] for NO, [6] for CMX, [22–24, 49] for AMX.

The Gibbs free energy is calculated for each catabolic reaction per mole of electron donor (Eqs. (1)–(5)). In the catabolic activities considered, no ATP is produced via substrate level phosphorylation therefore the energy for growth and maintenance comes solely from the membrane potential. The amount of energy harvested ($\Delta G_{Cat}^{01}$) is calculated according to Eq. (6).

6 $$\Delta G_{Cat}^{01} = v_A^ \ast \cdot \gamma _A^ \ast \cdot F\cdot \Delta \Psi$$

Where $v_A^ \ast$ is the amount of electron acceptor used in energy harvesting per mol of electron donor, $\gamma _A^ \ast$ is the number of electrons in each mole of electron acceptor, $F$ is the Faraday constant, and $\Delta \Psi$ is the potential difference invested in the energy conversion. $\Delta \Psi$ is calculated as the redox potential difference between the electron acceptor pair of the catabolic activity and the electron donor. For AO, NRMX and An-NRMX, $\Delta \Psi$ is calculated as the potential difference between the electron acceptor and the ubiquinone/ubiquinol pair ($\Delta \Psi = \Psi _A - \Psi _U$). Like this, it is considered that the energy-conversion steps are the oxidation of hydroxylamine to nitrite, specifically when the electrons are transferred from ubiquinone/ubiquinol pair to complex III (Q cycle) [28–30]. For NO activity, $\Delta \Psi$ is calculated as the potential difference between the electron acceptor (O_2_) and the electron donor (NO_2_^-^, $\Delta \Psi = \Psi _A - \Psi _D$) as the Q cycle does not participate in its catabolic process [31]. For CMX activity, $\Delta \Psi$ is calculated as the sum of the potential differences estimated for AO and NO activities. Like this it is considered that the enzymatic groups for ammonia oxidation and nitrite oxidation of comammox *Nitrospira* are fully compatible and catalyze their respective reactions and transport of electrons to complex IV without limiting the activity of the other enzymatic group.

The values of $\gamma _A^ \ast$ and reduction potentials at pH 7 ($\Psi _i$) used to calculate the $\Delta G_{Cat}^{01}$ values are shown in Supplementary Table S1. In those catabolic activities where ammonia oxidation takes place (AO, CMX, NRMX and An-NRMX), part of the consumed oxygen is used for ammonia activation in the monooxygenation step (1 mol of O_2_) where all the energy is considered dissipated [32].

For all the activities evaluated, the energy required to generate 1 mole of new biomass (CH_1.8_O_0.5_N_0.2_) from inorganic carbon was approximated using *The Dissipation method*, [33] which defines that the expensive mechanism of reversed electron transfer is necessary in autotrophic growth, estimating energetic requirements as a constant of 3500 kJ per C-mole of new biomass formed ($\Delta G_{Ana}^{01}$). The growth yield values ($Y_{X/D}$, in units of C-mole of biomass formed per mole of electron donor) are calculated according to Eq. (7). With the estimated $Y_{X/D}$ value and the overall stoichiometry of the catabolic activities considered (Eqs. (1)–(5)), the growth yield over electron acceptor and other metabolic compounds is also calculated.

7s $$Y_{X/D} = \Delta G_{Cat}^{01}/\Delta G_{Ana}^{01}$$

The calculated growth yields are compared with the available experimental values when this is possible (AO, NO and CMX metabolic activities) but also with those obtained applying the Thermodynamic Electron Equivalents revised Model (TEEM2) [34] (Table 1). The detailed description of the TEEM2 methodology can be found in Supplementary Methods S1.

The estimated growth yield values for AO and NO activities agreed with the experimental values reported. The growth yield value reported for CMX was 15% higher than the one predicted with our developed methodology. The highest growth yield values for ammonia (Y_X/NH3_), nitrite (Y_X/NO2_) and oxygen (Y_X/O2_) were found for CMX metabolism, AMX bacteria and NO metabolism respectively, tendencies that were also observed for the calculated theoretical values. Comparing the growth yields predicted by our methodology and the TEEM2, less than 10% of difference was found in the growth yield estimations for AO, NO and CMX (1.0%, 7.7% and 8.2%, respectively). The TEEM2 methodology also predicts the highest Y_X/NH3_, Y_X/NO2_ and Y_X/O2_ values for CMX, AMX and NO activities, respectively.

### Multiscale model to describe the community assembly of comammox *Nitrospira* and anammox bacteria

An Individual-based Model framework was employed to simulate the community assembly of comammox *Nitrospira* (considering the aforementioned metabolic activities) and anammox bacteria. These grew in a suspended floc system under a dynamic nitrogen and oxygen limited environment, simulating an ideal continuous stirred tank reactor until it reaches steady state. Reactor parameters were based on the experiment setup of van Kessel et al. study [5] (see Supplementary Table S2). The chemical species considered (ammonia, nitrite, nitrate, oxygen and carbon dioxide) diffused through the flocs and they were consumed or produced locally by the individuals. Function of the local environmental conditions, individuals would change their growth and decay rates. The model consists of two sub-models: (*i*) a *physical model* describing the diffusive transport of chemical species, and (*ii*) a *biological model* to simulate the growth and decay rate of each individual considering the heterogeneity of the system and the intrinsic ecological interactions between microbial species. The parameters of the model are presented in Supplementary Table S2. The *physical model* consists of five diffusion-reaction partial differential equations (2^nd^ Fick’s law plus reaction term) coupled with the local situation and information of microbial entities (mass, stoichiometries and kinetics). The diffusion-reaction equations were solved for each individual spatial grid, in which the 2D space was divided, through a multigrid method (see Supplementary Methods S2 – *Discretization of diffusion-reaction equation*).

Microbial growth is modeled following a Monod expression that describes the dependence of growth rate for an *n* individual ($\mu ^n$) on the local concentration of the limiting substrate $S$ and its substrate affinity ($K_S^n$). The specific maintenance rate ($a^n$) is evaluated as a negative growth rate [35, 36] (Eq. (8)). To describe the activity of anammox bacteria, oxygen inhibition is described by the addition of a non-competitive inhibition expression to Eq. (8), which is function of the oxygen concentration and the inhibition constant $K_I^n$ [24, 37]. The presence of certain capacity for oxygen protection of aerobic *Nitrospira* to anammox bacteria was ensured assuming $K_{I,O_2}^{AMX}/K_{O_2}^{Ns} = 1$, which is consistent with reported experimental data regarding the oxygen tolerance of anammox bacteria (Supplementary Table S3).

8 $$\mu ^n = \mu _{max}^n\cdot \prod \left( {\frac{{\left[ S \right]}}{{K_S^n + \left[ S \right]}}} \right)\cdot \frac{{K_{I,O_2}^n}}{{K_{I,O_2}^n + \left[ {O_2} \right]}} - a^n$$

In order to evaluate the actual influence of the metabolic heterogeneity on comammox *Nitrospira*, and to be able to extrapolate the outcome of this study, no particularities of specific bacteria species were considered. The kinetic parameters defining the Monod curve for comammox *Nitrospira* and anammox bacteria (*µ*_*max*_, K_N_, K_O2_, $a$) were assumed equal (non-kinetic competition, Supplementary Table S4) and were chosen following the guidelines stated below. Under strongly limited environmental conditions like the ones simulated, the competition for substrate has to be evaluated function of differences in growth yield (Y_X/S_), as growth occurs far from its maximum (*µ*_*max*_) [38]. Although differences in substrate affinity between species can play a role in selection (see Supplementary Results/Discussion), the reported range of affinity values of ammonia and nitrite for comammox *Nitrospira* and anammox bacteria overlaps (Supplementary Fig. S1). Therefore, ammonia and nitrite affinities (K_NH3_ and K_NO2_, respectively) are considered of 1.0 µM for all the simulations in agreement with the values reported for anammox bacteria (Supplementary Fig. S1). Lastly, complete metabolic stoichiometries of *Nitrospira* and anammox populations are detailed in Supplementary Table S5. We did not consider active movement, dispersion/invasion nor loss of microorganisms with effluent, but microbes move passively due to the shoving forces exerted by neighboring individuals as they grow and divide (see Supplementary Methods S2).

### Simulation experiments

All the simulation experiments started by considering 12 different microbial inocula randomly generated. The time of simulation was 5.0 years based on the early enrichments of comammox *Nitrospira* [4, 5], obtaining a stable community of comammox *Nitrospira* and anammox bacteria. The concentration of substrates and products in the liquid bulk were calculated according to the mass balance of a reactor operating in a continuous mode, where the simulated activity was assumed representative of the average activity of the whole reactor (see Supplementary Methods S2 – *Calculus of bulk liquid concentrations*).

The environmental conditions were selected based on early studies of comammox *Nitrospira* [4, 5]. Three different nitrogen feeding regimes were applied (defined as NH_3_:NO_2_:NO_3_): ammonia feeding – 500:0:0 µM [4]; equimolar feeding – 500:500:500 µM [5]; and non-equimolar feeding – 500:375:500 µM [5]. The influence of oxygen availability was evaluated by establishing a constant bulk liquid oxygen concentration: anaerobic/hypoxic conditions (0.0 µM, 1.0 µM, 1.5 µM and 3.0 µM of O_2_) [5], and aerobic conditions (93.8 µM of O_2_) [4]. For each condition, the simulations were run in triplicates using different inocula (randomly generated).

Two additional sets of simulation experiments were performed to complement the main results of this work. The first one considering a population of comammox *Nitrospira* exclusively on CMX activity to assess the importance of metabolic heterogeneity in its survival under hypoxic conditions. The second one with lower and higher feedings of ammonia in aerobic conditions (93.8 µM of O_2_) following the experiment presented in Daims et al. (2015) [4].

### Ecological analysis at floc level

We performed an ecological analysis at floc level through Kendall’s correlation rank test on relative abundances of comammox *Nitrospira* activities (AO, NO, CMX, NRMX and An-NRMX) and anammox bacteria (AMX). The Kendall’s correlation coefficient (Kendall’s τ) ranges from –1 to +1. A value of –1 indicates that one dataset ranking is reversed (i.e., negative correlation), whereas a value of +1 indicates that the two rankings of the datasets are the same (i.e., positive correlation). A value of 0 indicates no correlation between the datasets. The relative abundances of each metabolic pair were collected independently from all aggregates and replicates before using them in the correlation test. Only those aggregates in which both metabolisms were present in the inoculum were subjected to the pairwise Kendall’s rank test. The interpretation of Kendall’s τ from an ecological perspective depends on the type of ecological interaction:

9 $${{{{{{{\mathrm{Collaboration}}}}}}}} \Rightarrow \left\{ {\begin{array}{*{20}{c}} {\tau \, > \, 0 \to + {{{{{{{\mathrm{influence}}}}}}}}} \\ {\tau = 0 \to {{{{{{{\mathrm{no}}}}}}}}\,{{{{{{{\mathrm{influence}}}}}}}}} \end{array}} \right.$$

10 $${{{{{{{\mathrm{Competition}}}}}}}} \Rightarrow \left\{ {\begin{array}{*{20}{c}} {\tau = 0 \to {{{{{{{\mathrm{no}}}}}}}}\,{{{{{{{\mathrm{influence}}}}}}}}} \\ {\tau \, < \, 0 \to - {{{{{{{\mathrm{influence}}}}}}}}} \end{array}} \right.$$

11 $$\begin{array}{*{20}{c}} {{{{{{{{\mathrm{Collaboration}}}}}}}}} \\ + \\ {{{{{{{{\mathrm{Competition}}}}}}}}} \end{array} \Rightarrow \left\{ {\begin{array}{*{20}{c}} {\tau \, > \, 0 \to + {{{{{{{\mathrm{influence}}}}}}}}} \\ {\tau = 0 \to {{{{{{{\mathrm{no}}}}}}}}\,{{{{{{{\mathrm{influence}}}}}}}}} \\ {\tau \, < \, 0 \to - {{{{{{{\mathrm{influence}}}}}}}}} \end{array}} \right.$$

Commensalism and syntrophic interactions are considered as collaboration. Kendall’s correlation rank test was performed with MATLAB (R2020b) via the built-in function ‘corr()’.

### Parameters for the quantification of nitrogen removal

In this study, the amount of ammonia and nitrite oxidized to nitrate is referred as aerobic nitrification performance, and calculated by Eq. (12).

12 $$Aerobic\,nitrification\,performance\left( {{{{{{{\mathrm{\% }}}}}}}} \right) = \frac{{\left[ {NO_3^ - } \right]_{net}}}{{\left[ {NH_3} \right]_{inf} + \left[ {NO_2^ - } \right]_{inf}}}\cdot 100$$

Where $\left[ {NO_3^ - } \right]_{net}$ is the net concentration of nitrate in the bulk liquid (difference between concentration in bulk liquid and influent, $\left[ {NO_3^ - } \right]_{BL} - \left[ {NO_3^ - } \right]_{inf}$), $\left[ {NH_3} \right]_{inf}$. is the concentration of ammonia in the influent, and $\left[ {NO_2^ - } \right]_{inf}$ is the concentration of nitrite in the influent. The rest of ammonia and nitrite is oxidized to N_2_ by anaerobic activities (An-NRMX and AMX), Eq. (13).

13 $$Anaerobic\,oxidation\,performance\left( {{{{{{{\mathrm{\% }}}}}}}} \right) = \left( {1 - \frac{{\left[ {NO_3^ - } \right]_{net} - \left\{ {\left[ {NH_3} \right]_{BL} + \left[ {NO_2^ - } \right]_{BL}} \right\}}}{{\left[ {NH_3} \right]_{inf} + \left[ {NO_2^ - } \right]_{inf}}}} \right)\cdot 100$$

Where $\left[ {NH_3} \right]_{BL}$ and $\left[ {NO_2^ - } \right]_{BL}$ are ammonia and nitrite concentration in bulk liquid, respectively.

### Statistical analyses

The statistical significance of the differences between relative abundances (wt. %) and metabolic ratios across the nitrogen feeding regimes and oxygen concentrations, were assessed using the Welch’s *t*-test [39]. To evaluate the correlation among the relative abundances of *Nitrospira*’s metabolic activities and oxygen concentration, Kendall’s rank correlation method (τ) was employed [40].

## Results

**Fig. 1:**
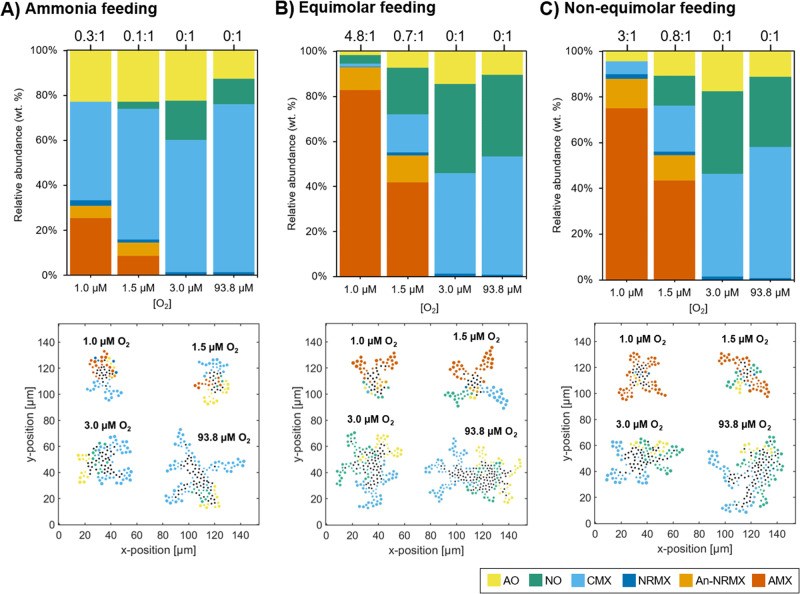
Influence of nitrogen feeding regime (defined by NH_3_:NO_2_:NO_3_ ratio) and oxygen concentration (1.0, 1.5, 3.0 and 93.8 µM) on comammox Nitrospira and anammox bacteria community. Relative abundances of *Nitrospira* metabolic activities (AO, NO, CMX, NRMX and An-NRMX) and anammox bacteria (AMX), with each correspondent floc images (bottom panels) under **A** ammonia feeding (NH_3_:NO_2_:NO_3_ = 500:0:0 µM), **B** equimolar feeding (NH_3_:NO_2_:NO_3_ = 500:500:500 µM), and **C** non-equimolar feeding (NH_3_:NO_2_:NO_3_ = 500:375:500 µM). Labels over each bar show Anammox:*Nitrospira* ratio at steady state. The statistical significance between different oxygen concentrations and nitrogen feeding regimes are shown in Supplementary Tables S7 and S8, respectively. Black circles on floc images represent inactive individuals. Additional floc images of comammox *Nitrospira* and anammox community are shown in the Supplementary Fig S2.

Figure 1 shows the results from the simulation experiments of comammox *Nitrospira* and anammox bacteria community growing on suspended flocs under nitrogen and oxygen limiting conditions. Three different nitrogen feeding regimes (defined by NH_3_:NO_2_:NO_3_ ratio) and five oxygen concentrations were evaluated [4, 5]. All simulation experiments started with same proportion of comammox *Nitrospira* performing one of the mentioned metabolic activities (AO, NO, CMX, NRMX or An-NRMX) and anammox bacteria.

In all tested conditions, the co-existence of different metabolic activities of comammox *Nitrospira* was observed in steady state, predicting metabolic heterogeneity for comammox *Nitrospira* in limiting conditions of nitrogen and oxygen. Only two metabolic activities were totally suppressed under specific conditions: (*i*) NO activity at 1.0 µM of O_2_ and only feeding of ammonia or non-equimolar nitrogen regime (Fig. 1A, C), and (ii) An-NRMX activity at ≥ 3.0 µM of O_2_ regardless of the nitrogen feeding regime. Although NRMX activity was not completely suppressed, its presence on the community was always under 2.0 wt. %. In general, no specific distribution of comammox *Nitrospira* or anammox bacteria was observed (Fig. 1 and Supplementary Fig. S2). The absence of stratification of aerobic comammox *Nitrospira* (performing AO, NO, CMX or NRMX) and anammox bacteria suggested that anammox did not need to be protected against oxygen at these low oxygen concentrations (≤1.5 µM of O_2_). Stratification of AO and NO activities (an expected distribution in commensalism) was only observed when ammonia feeding and aerobic conditions (93.8 µM of O_2_) were applied (Fig. 1A and Supplementary Fig. S2A).

In anaerobic conditions, AMX dominated the community in both equimolar and non-equimolar feedings of ammonia and nitrite (~96 wt. % of AMX and ~4 wt. % of An-NRMX). Anaerobic activities (An-NRMX and AMX) only remained active at 1.5 µM of O_2_ or below (Fig. 1). In these hypoxic conditions, aerobic comammox *Nitrospira* (performing AO, NO, CMX or NRMX) were also present. The aerobic activities always outnumbered the anaerobic ones when ammonia feeding was applied (69.1% of aerobes and 30.9% of anaerobes at 1.0 µM of O_2_, *p* < 0.003; 85.3% of aerobes and 14.7% of anaerobes at 1.5 µM of O_2_, *p* < 0.001; Fig. 1A). NO activity was significantly benefited by an equimolar feeding of ammonia and nitrite (*p* < 0.01; Fig. 1B), turning to be the dominant aerobic activity at 1.0 µM of O_2_. A significant reduction of NO abundance (or a total suppression at 1.0 µM of O_2_) was obtained with the reduction of nitrite in the feeding and the presence of anammox bacteria (Fig. 1A, C). At higher concentrations of oxygen (≥3.0 µM of O_2_), ammonia and nitrite oxidation were mainly carried out by AO, NO and CMX activities (~98 wt. %) in all feeding regimes. Although we did not set any oxygen inhibition to An-NRMX activity, this metabolic activity was totally supressed by their competitors.

The coexistence of division of labor (AO + NO) and complete ammonia oxidation was present almost in all cases except in those conditions where NO activity was totally suppressed (Fig. 1A, C at 1.0 µM of O_2_). Absolute dominance of CMX activity over division of labor was not observed in any case. Applying ammonia feeding only (Fig. 1A), CMX activity significantly outnumbered division of labor, increasing their dominance at higher oxygen concentration (23% of AO + NO and 44% of CMX at 1.0 µM of O_2_, *p* < 0.04; 26% of AO + NO and 58% of CMX at 1.5 µM of O_2_, *p* < 0.009; 40% of AO + NO and 59% of CMX at 3.0 µM of O_2_, *p* < 0.001; 24% of AO + NO and 75% of CMX at 93.8 µM of O_2_, *p* < 0.001; Supplementary Fig. S3A). Applying equimolar feeding (Fig. 1B), division of labor significantly outnumbered CMX activity under hypoxic conditions (5% of AO + NO and 1% of CMX at 1.0 µM of O_2_, *p* < 0.03; 28% of AO + NO and 17% of CMX at 1.5 µM of O_2_, *p* < 0.05; 54% of AO + NO and 45% of CMX at 3.0 µM of O_2_, *p* < 0.002; 47% of AO + NO and 52% of CMX at 93.8 µM of O_2_, *p* = 0.238; Fig. S3A). Same proportion of division of labor and CMX activity was always observed in non-equimolar feeding (*p* > 0.08; Fig. 1C). Although AO activity never outnumbered CMX, a correlation between lower oxygen concentrations and higher relative proportions of AO over CMX was observed (*τ* = 1.0, *p* < 0.02 for only ammonia feeding; *τ* = 0.8, *p* < 0.05 for equimolar feeding; *τ* = 0.8, *p* < 0.05 for non-equimolar feeding; Supplementary Fig. S3B).

## Discussion

Comammox *Nitrospira* cocultured with anammox bacteria [9, 10] has the potential to achieve high levels of nitrogen removal with a reduced energy consumption for aeration, limited N_2_O emissions [41], and sludge production [42]. However, its activity remains unexplained, including their capacity to survive in hypoxic conditions and their actual contribution to biological nitrogen removal. Using a multiscale model (Individual-based Model), we have shown that under oxygen and/or nitrogen limiting conditions, selective co-existence of different metabolic activities of comammox *Nitrospira* occurs (metabolic heterogeneity). Our modeling results suggest that even at extremely low oxygen concentrations (1.0 µM of O_2_) comammox *Nitrospira* is able to survive in a proportion similar to the experimentally observed in van Kessel et al. (2015) [5] (Anammox:*Nitrospira* ≈ 3:1).

Complete dominance of any of the metabolic activities of comammox *Nitrospira* was not observed at steady state for any simulated conditions. The trends observed in the relative abundances associated to NO and CMX activities can be explained by the metabolic efficiencies associated to these activities (growth yield values of Table 1). NO activity dominated as aerobic activity under oxygen limitation when nitrite was available (Fig. 1B; 1 µM of O_2_). This can be explained because it is the activity with the highest growth yield on oxygen (Y_X/O2_). CMX activity dominated when ammonia was limited and oxygen not (Fig. 1A; 93.8 µM of O_2_) due to its higher efficiency on ammonia (Y_X/NH3_) over its aerobic competitors.

Under strong limited conditions of substrates and high competition, metabolic efficiency defines the survival of microbial species. In low oxygen conditions, comammox *Nitrospira* performing NO activity was outcompeted by AMX when nitrite feeding was withdrawn or reduced (Fig. 1A, C; 1 µM of O_2_), because AMX has a higher growth yield on nitrite (Y_X/NO2_) than NO populations (Table 1). Repression of NO benefited the other metabolic activities of comammox *Nitrospira* with lower Y_X/O2_ (AO, CMX and NRMX). Under this competitive environment, the collaboration between comammox *Nitrospira* (performing AO or NRMX) and anammox bacteria emerges (collaborative competition, Fig. 1A, C). In all tested conditions AO, CMX and NRMX activities coexisted. Although the growth yield of CMX on ammonia (Y_X/NH3_) and oxygen (Y_X/O2_) was higher than AO and NRMX, the difference was not enough for the suppression of the latter activities.

### Metabolic heterogeneity for comammox *Nitrospira*

**Fig. 2:**
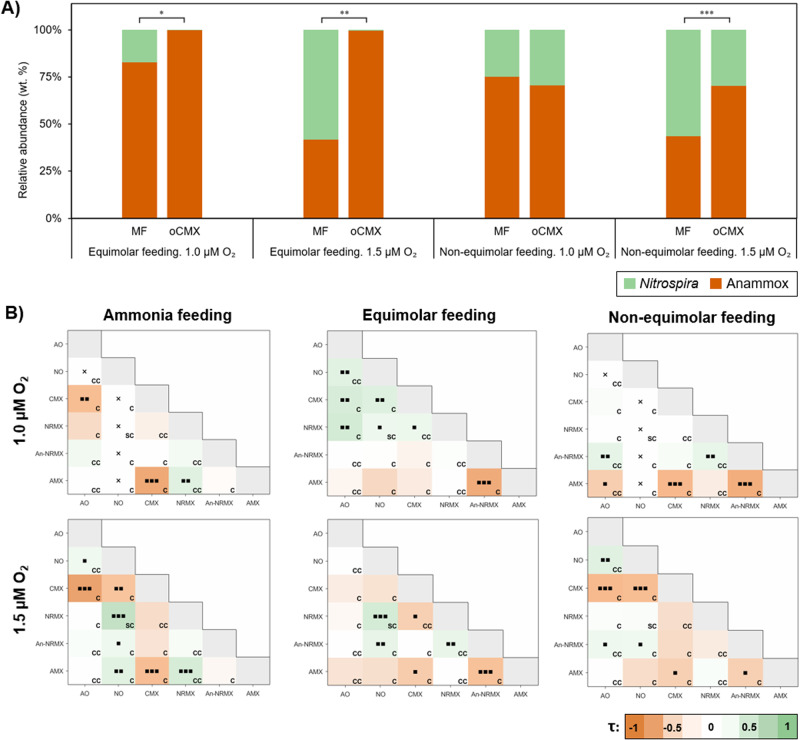
Value of metabolic heterogeneity for comammox Nitrospira. **A** Impact of metabolic heterogeneity on the survival of comammox *Nitrospira* under hypoxic conditions (1.0 and 1.5 µM of O_2_) applying equimolar feeding (NH_3_:NO_2_:NO_3_ = 500:500:500 µM) and non-equimolar feeding (NH_3_:NO_2_:NO_3_ = 500:375:500 µM). MF – simulation experiments considering metabolic flexibility in *Nitrospira*; oCMX – simulation experiments considering that *Nitrospira* only performs CMX activity. Asterisks denote *p*-value significance where *p < 0.05; **p < 0.01; ***p < 0.001. **B** Ecological analysis at floc level under conditions where comammox *Nitrospira* and anammox bacteria remained active (1.0 and 1.5 µM of O_2_). Ammonia feeding (NH_3_:NO_2_:NO_3_ = 500:0:0 µM, left panels); equimolar feeding (NH_3_:NO_2_:NO_3_ = 500:500:500 µM, center panels); non-equimolar feeding (NH_3_:NO_2_:NO_3_ = 500:500:500 µM, right panels). Kendall’s τ values of metabolisms are presented on a color scale. Dotted cells indicate *p*-value significance where ▪*p* < 0.05; ▪▪*p* < 0.01; ▪▪▪*p* < 0.001. Cross symbol (x) indicates no co-existence of the metabolic pair at the end of the simulation experiments. Bottom-right labels indicate the ecological interaction of metabolic pair: CC – Commensalism + Competition; SC – Syntrophism + Competition; C – Competition. Sample sizes employed for Kendall’s τ calculation are shown in Supplementary Fig. S5.

Our results suggest that comammox *Nitrospira* might only be able to thrive together with anammox bacteria under hypoxic conditions because of its metabolic heterogeneity (Fig. 2A). This is highlighted in the cases in which anammox bacteria are not dependent on other individuals for nitrite availability (equimolar feeding case; Fig. 2A). Additionally, metabolic heterogeneity would explain the ubiquity of comammox *Nitrospira* and their stable association with anammox bacteria. The considered simple competition among comammox *Nitrospira* and anammox bacteria becomes a more complex ecological network, which combines both collaborative interactions (commensalism and syntrophism) and competition (Fig. 2B).

The influence of ecological interactions on individuals (represented with Kendall’s τ; see Methods – *Ecological analysis at floc level*) was significantly positive (Kendall’s τ > 0) or no influence was detected (Fig. 2B). This evidences the positive impact of metabolic heterogeneity for comammox *Nitrospira*. Note that positive influence was also observed in the competition cases (C label in Fig. 2B). This is because a third activity favored both competitive metabolic activities with a commensal interaction (higher-order interactions; e.g., AO, NO and An-NRMX: AO activity fed nitrite to both NO and An-NRMX activities). Note that with an equimolar feeding and 1.0 µM of O_2_ (1^st^ row and 2^nd^ column; Fig. 2B), positive interaction among all aerobic activities of comammox *Nitrospira* (AO, NO, CMX, NRMX) was observed. The strong hypoxic conditions and feeding of nitrite give a clear advantage of anaerobic activities (An-NRMX and AMX) over the aerobic ones (AO, NO, CMX, NRMX). However, some individuals performing aerobic activities were able to survive because the difference of Y_X/O2_ was not enough to supress any aerobic activity by oxygen competition. Because all aerobic individuals were strongly constrained by low oxygen concentration, the acquisition of space by any aerobic activity gave an opportunity to the anaerobic individuals to take the space (i.e., An-NRMX and AMX). In this case, space competition (instead of competition for substrate) controlled the community assembly – less competitive individuals (aerobic activities) versus more competitive individuals (anaerobic activities).

In those conditions where anammox bacteria were not present (≥3.0 µM of O_2_; Supplementary Fig. S4) a strong competition for oxygen between CMX and NO activities was still present. The restriction of NRMX by CMX activity (due to the lower metabolic efficiency for ammonia of NRMX, Table 1) and the uniform influence of interactions in all feeding regimes were only observed in aerobic conditions (Supplementary Fig. S4, 93.8 µM of O_2_). Throughout the tested conditions (Fig. 2B and Supplementary Fig. S4), a variety of Kendall’s τ values was observed, indicating the adaptability that metabolic heterogeneity gives to comammox *Nitrospira*.

The collaboration between comammox *Nitrospira* and anammox bacteria is supported by AO and NRMX activities, yielding nitrite for anammox bacteria. Figure 2B shows that comammox *Nitrospira* performing NRMX would be a better partner for anammox bacteria than when performing AO activity (Kendall’s τ ≥ 0 between AMX and NRMX; Kendall’s τ ≤ 0 between AMX and AO, last row of panels in Fig. 2B). The theoretically predicted lower growth yield for ammonia of NRMX activity reduces the competitive pressure to anammox bacteria and increases the production of nitrite per biomass generated.

### Optimization of anaerobic oxidation performance

**Fig. 3:**
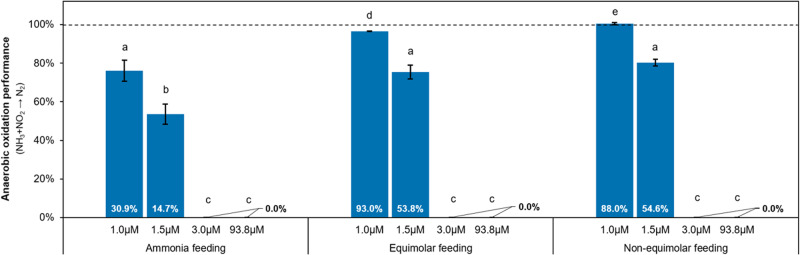
Influence of nitrogen feeding regime (ammonia, equimolar and non-equimolar feeding) and oxygen concentration (1.0, 1.5, 3.0 and 93.8 µM) on the anaerobic oxidation performance (ammonia and nitrite oxidation to N_2_) expressed as percentage. Data labels depict the relative abundance of anaerobic activities (An-NRMX and AMX). Nitrogen feeing regimes: ammonia feeding – 500:0:0 µM; equimolar feeding – 500:500:500 µM; non-equimolar feeding – 500:375:500 µM. Error bars show standard deviation of *n* = 3 simulation replicates. Bars that do not share similar letters denote statistical significance, *p* < 0.05. For more information about the calculus of the anaerobic oxidation performance (see Methods – *Parameters for the quantification of nitrogen removal*).

The *in-silico* experiments show that NRMX activity has the capacity to positively contribute to the collaboration between comammox *Nitrospira* and anammox bacteria by reducing the nitrate produced by CMX and NO activities to nitrite (always Kendall’s τ ≥ 0 between NRMX and AMX; Fig. 2B). Considering this, the collaboration between *Nitrospira* and anammox was *in-silico* maximized by (*i*) reducing the oxygen concentration to minimize the inhibition of anammox bacteria, and by (*ii*) feeding ammonia and nitrite non-equimolarly (1:0.75) ensuring the suppression of NO activity responsible for the drain of nitrite (Fig. 3).

Furthermore, improvement of anaerobic oxidation performance was predicted, showing similar performance with lower relative abundance of anaerobic activities (see data labels of statistic group *a*, or comparison between group *d* and *e* in Fig. 3). Due to the low metabolic efficiency of NRMX with respect to ammonia (low Y_X/NH3_ value, Table 1), the collaboration between comammox *Nitrospira* and anammox bacteria was also predicted as dependent on the residual nitrate concentration (lower anaerobic oxidation performance was observed when non-equimolar feeding without nitrate was applied, *p* < 0.05; Supplementary Fig. S6).

### Metabolic heterogeneity explains transient accumulation of nitrite

**Fig. 4:**
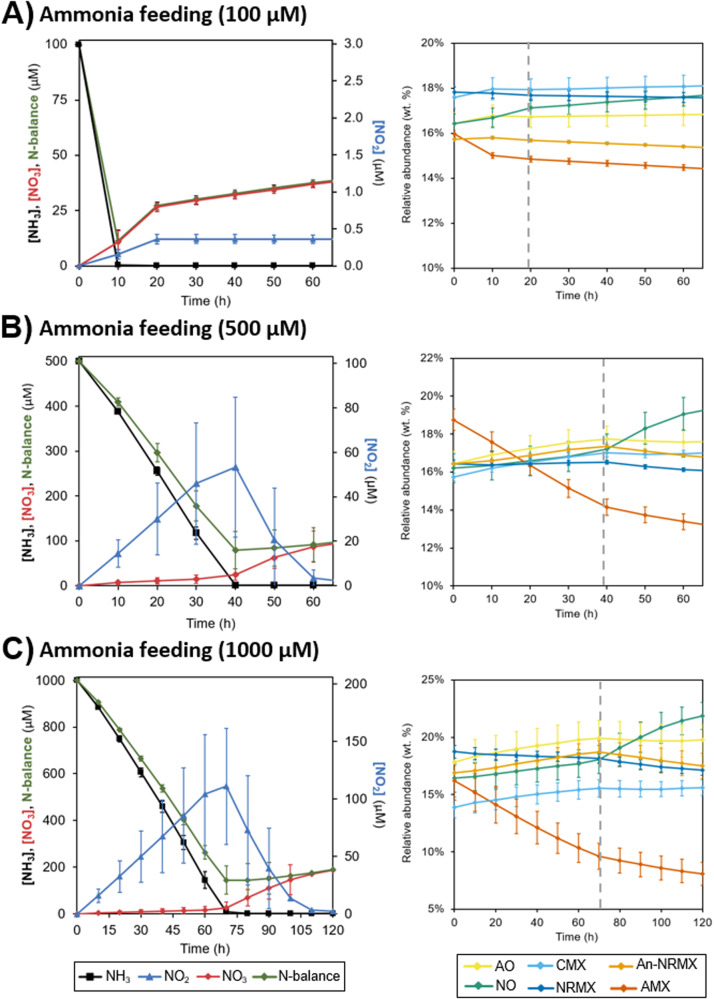
Influence of ammonia concentration to the transient accumulation of nitrite at 93.8 µM of O_2_. Three ammonia concentrations were tested. Only 60–120 h are shown (full simulation in Supplementary Fig. S7): 100 µM of NH_3_ (panels **A**), 500 µM of NH_3_ (panels **B**), and 1000 µM of NH_3_ (panels **C**). Left panels show the dynamics of nitrogen compounds (NH_3_, NO_2_ and NO_3_) at the early stages of simulation. Right panels show the evolution of relative abundances of comammox *Nitrospira* and anammox bacteria. Error bars show standard deviation of *n* = 3 simulation replicates. If not visible, error bars are smaller than symbols. Gray dashed lines in right panels (dynamics of metabolic activities) depict the time with the maximum concentration of nitrite.

Daims et al. (2015) observed a transient accumulation of nitrite correlating with different ammonia feedings. With the objective to validate the model, and mechanistically explain the transient accumulation of nitrite, two extra sets of simulation experiments were developed with different ammonia concentrations and aerobic conditions (100 µM and 1000 µM of NH_3_ at 93.8 µM of O_2_; Fig. 4A, C, respectively). The same trend as the experimental findings was observed in the *in-silico* experiments: no transient accumulation of nitrite at low ammonia concentration (Fig. 3A), and a larger peak of nitrite transient accumulation at increasing ammonia concentrations (53.4 ± 31.5 µM of NO_2_ for 500 µM of NH_3_; 110.9 ± 33.2 µM of NO_2_ for 1000 µM of NH_3_; *p* < 0.05, *n* = 3; Fig. 4B, C, respectively). The coexistence of division of labor (AO + NO) and CMX activity together with the dissociation between AO and NO activities can explain the transient accumulation of nitrite in the bulk liquid (significant lag phase of NO activity was observed at 500 µM and 1000 µM of NH_3_; Fig. 4B, C).

## Conclusions

Based on the state-of-the-art knowledge of comammox *Nitrospira* and a bioenergetics analysis, the *in-silico* experiments presented in this study predict that metabolic heterogeneity for comammox *Nitrospira* is necessary for its survival under oxygen limiting environments. Spatial transcriptomics would be needed to fully confirm our in-silico findings (e.g., parallel-sequential fluorescence in situ hybridization; par-seqFISH [17]), but according to our simulation experiments, metabolic heterogeneity is a mechanistic explanation of the early findings on comammox *Nitrospira* – its co-existence with anammox bacteria under hypoxic conditions [5], the dominance of complete nitrification activity in nitrogen limiting environments, and the transient accumulation of nitrite under aerobic conditions [4]. When availability of nitrite is lower than ammonia, this work predicts that maximization of anaerobic nitrogen removal by anammox bacteria is possible by supressing comammox *Nitrospira* performing NO activity.

### Supplementary information

Supplementary information

## Data Availability

The data that supports the findings of this study are available in the Supplementary Material of this article. The source code used to produce the results and analyses presented in this study are available on a public GitHub repository at https://github.com/Computational-Platform-IbM/IbM.
