# Supplementary material for: Competitive and substrate limited environments drive metabolic heterogeneity for comammox Nitrospira
Source: ISME Commun. 2023 Aug 29;3:91. doi: 10.1038/s43705-023-00288-8 (PMC10465561; doi:10.1038/s43705-023-00288-8)
Supplement: Supplementary file 1 — Supplementary information [file 43705_2023_288_MOESM1_ESM.docx]

**Supplementary Information**

**Competitive and substrate limited environments drive metabolic heterogeneity for comammox *Nitrospira***

Eloi Martinez-Rabert^1,2^, Cindy J. Smith^1^, William T. Sloan^1^, Rebeca Gonzalez-Cabaleiro^2✉^

^1^James Watt School of Engineering, Infrastructure and Environment Research Division, University of Glasgow, Advanced Research Centre, Glasgow, United Kingdom

^2^Department of Biotechnology, Delft University of Technology, Delft, Netherlands

^✉^Corresponding author. E-mail: [r.gonzalezcabaleiro@tudelft.nl](mailto:r.gonzalezcabaleiro@tudelft.nl)

**Contents**

[Supplementary Methods S1 – Calculation of growth yield through TEEM2 2](#_Toc136984498)

[Supplementary Methods S2 – Detailed description of mathematical model 4](#_Toc136984499)

[Discretization of diffusion-reaction equation 4](#_Toc136984500)

[Boundary conditions of simulation domain 5](#_Toc136984501)

[Calculus of bulk liquid concentrations 6](#_Toc136984502)

[Domain definition – diffusion and no-diffusion regions 6](#_Toc136984503)

[Reaction term from microbial activity 9](#_Toc136984504)

[Shoving algorithm 10](#_Toc136984505)

[Integration 12](#_Toc136984506)

[Summary of model boundary, range of application and model assumptions 14](#_Toc136984507)

[Supplementary Results/Discussion – (Eco)physiological analysis of comammox *Nitrospira* 16](#_Toc136984508)

[Supplementary Figures 18](#_Toc136984509)

[Supplementary Tables 27](#_Toc136984510)

[References 38](#_Toc136984511)

**Abbreviations**

**AOB** – Ammonia oxidizing bacteria; **NOB** – Nitrite oxidizing bacteria; **AMX** – anammox bacteria (performing anaerobic ammonia oxidation); **AO** – Ammonia oxidation (catabolism); **NO** – Nitrite oxidation (catabolism); **CMX** – Complete ammonia oxidation (catabolism); **NRMX** – Nitrate-reducing ammonia oxidation (catabolism); **An-NRMX** – Anaerobic nitrite-reducing ammonia oxidation (catabolism).

# Supplementary Methods S1 – Calculation of growth yield through TEEM2

We compared our results with the growth yield calculated through Thermodynamic Electron Equivalents revised Model (TEEM2) (ref. 1). TEEM2 is a generalized method for the estimation of the growth yield of any microbial functional group based on thermodynamics. The balance between anabolism and catabolism is closed using electron equivalent units (eeq) and specific assumptions related with the necessary energy to growth.

TEEM2 considers the use of half oxidoreductive reactions for electron donor (eD) and electron acceptor (eA). In this case, all comammox *Nitrospira* metabolisms are based on three half reactions of eD ($NH_{4}^{+}\to NO_{2}^{-}$, $NO_{2}^{-}\to NO_{3}^{-}$, $NH_{4}^{+}\to NO_{3}^{-}$; Eqs. S1 – S3, respectively), and three half reactions of eA ($O_{2}\to H_{2}O$, $NO_{3}^{-}\to NO_{2}^{-}$, $NO_{2}^{-}\to NO$; Eqs. S4 – S6, respectively). The methods for developing all half reaction and the Gibbs energy of formation ($\Delta G_{f}^{o'}$) of each chemical component are provided by Rittmann and McCarty (2020) (ref. 2).

$\frac{1}{6}NH_{4}^{+}+\frac{1}{3}H_{2}O\to\frac{1}{6}NO_{2}^{-}+\frac{4}{3}H^{+}+e^{-} \Delta G_{d}^{o'}=32.928 kJ/eeq$ (S1)

$\frac{1}{2}NO_{2}^{-}+\frac{1}{2}H_{2}O\to\frac{1}{2}NO_{3}^{-}+H^{+}+e^{-} \Delta G_{d}^{o'}=41.649 kJ/eeq$ (S2)

$\frac{1}{8}NH_{4}^{+}+\frac{3}{8}H_{2}O\to\frac{1}{8}NO_{3}^{-}+\frac{5}{4}H^{+}+e^{-} \Delta G_{d}^{o'}=35.108 kJ/eeq$ (S3)

$\frac{1}{4}O_{2}+H^{+}+e^{-}\to\frac{1}{2}H_{2}O \Delta G_{a}^{o'}=-78.719 kJ/eeq$ (S4)

$\frac{1}{2}NO_{3}^{-}+H^{+}+e^{-}\to\frac{1}{2}NO_{2}^{-}+\frac{1}{2}H_{2}O \Delta G_{a}^{o'}=-41.649 kJ/eeq$ (S5)

$\frac{1}{2}NO_{2}^{-}+H^{+}+e^{-}\to\frac{1}{2}NO+\frac{1}{2}H_{2}O \Delta G_{a}^{o'}=-9.110 kJ/eeq$ (S6)

For each metabolism of comammox *Nitrospira*, the energy conversion in the catabolism ($\Delta G_{r}$, Eq. S7) is determined from the half reaction reduction potentials for the eA ($\Delta G_{a}^{o'}$) and eD ($\Delta G_{d}^{o'}$). One of the modifications involved in TEEM2 is the consideration that oxygenase reactions generally require the input of energy and reducing power in the form of NADH. This is represented by the difference between $\Delta G_{a}^{o'}$ of oxygen (-78.72 kJ/eeq) and $\Delta G_{d}^{o'}$ of the NADH/NAD^+^ half reduction equation (30.88 kJ/eeq), which equals to –109.6 kJ/eeq (ref. 1). In this case, 2/6 of electrons from ammonia are employed in its oxygenase step, which means that the actual energy loss is ‑36.52 kJ/eeq. Thus, the energy loss in kJ per mole of donor is included in Eq. S7, where $q$ is the number of times an oxygenase is used in the complete oxidation of the respective eD.

$\Delta G_{r}=\Delta G_{a}^{o'}-\Delta G_{d}^{o^{'}}-q\cdot\Delta G_{xy}$ (S7)

Half reactions for electron donor and cell synthesis are combined to produce the synthesis reaction, referred here as Gibbs free energy for synthesis ($\Delta G_{S}$). In TEEM2, $\Delta G_{S}$ is evaluated by Eq. S8.

$\Delta G_{S}=\frac{\Delta G_{fa}-\Delta G_{d}}{\epsilon^{m}}+\frac{\Delta G_{in}-\Delta G_{fa}}{\epsilon^{n}}+\frac{\Delta G_{pc}}{\epsilon}$ (S8)

Where $\Delta G_{fa}$ is the Gibbs free energy of the half reaction for formaldehyde (46.53 kJ/eeq), $\Delta G_{in}$ is the Gibbs free energy of the half reaction of intermediate synthesis (30.9 kJ/eeq), and $\Delta G_{pc}$ is the Gibbs free energy for cell synthesis (18.8 kJ/eeq, assuming the cell relative composition of C_5_H_7_O_2_N and ammonia as the source for cell synthesis). $m$ and $n$ are two adjustments that are set as follows: $m$ equals 1 for C1 compounds (as CO_2_) and equals $n$ for all others; the exponent $n$ equals +1 if $m=n$ and $\left( \Delta G_{in}-\Delta G_{d} \right)>0$, otherwise it equals -1. The overall reaction for cell growth is obtained by combining in proper proportion (represented by $A$, Eq. S9) the synthesis reaction and energy reaction, considering that only part of the energy reaction will be employed for the synthesis of new biomass (energy-transfer efficiency, $\epsilon$). Finally, the growth yield is determined from A expressed in eeq units (represented by $f_{S}^{o}$, Eq. S10) or in molar units (represented by $Y_{X/D}$, Eq. S11).

$A=-\frac{\Delta G_{S}}{(\epsilon\cdot\Delta G_{r})}$ (S9)

$f_{S}^{o}=\frac{1}{(1+A)}$ (S10)

$Y_{X/D}={\left( \frac{\gamma_{D}}{\gamma_{X}} \right)\cdot f}_{S}^{o}$ (S11)

Where $\gamma_{D}$ is the degree of reduction of electron donor (6 eeq/mol_NH3_ for AO, NRMX and An‑NRMX; 2 eeq/mol_NO2_ for NO; 8 eeq/mol_NH3_ for CMX) and $\gamma_{X}$ is the degree of redaction of biomass (20 eeq/mol_X_), respectively. The energy-transfer efficiency ($\epsilon$) is a key factor that needs to be assumed or calibrated to solve Eq. S9 (ref. 1). The reported growth yield values of pure (or enriched) culture of AOB, NOB and CMX were used to calibrate $\epsilon$ for aerobic nitrifiers. For this, the Solver tool of Microsoft Excel (selecting GRG Nonlinear method) was employed. The average $\epsilon$ value found for aerobic nitrifiers (AOB, NOB and CMX) was 0.258 ± 0.040 (*n* = 13; see Table S6). This $\epsilon$ value was used to estimate the growth yields of comammox *Nitrospira* metabolisms (Table 1; Method section in main manuscript).

# Supplementary Methods S2 – Detailed description of mathematical model

## Discretization of diffusion-reaction equation

To solve the diffusion-reaction equation (Eq. S12), the implicit Crank-Nicolson method, which is unconditionally stable (3), is used to discretize in time the diffusion term. For the reaction term, an explicit forward Euler formula is used (Eq. S13). This can be done because the reaction process has a slower time scale than the diffusion (4, 5).

$\frac{\partial}{\partial t}\phi(x, y, t)=\mathbb{D\cdot}\nabla_{xy}^{2}\phi(x, y, t)+R(x, y, t)$ (S12)

$\frac{\phi_{i,j}^{n+1} - \phi_{i,j}^{n}}{h_{t}}\mathbb{=D\cdot}\frac{1}{2}\left[ \nabla^{2}\phi_{i,j}^{n+1}+\nabla^{2}\phi_{i,j}^{n} \right]+R(\phi_{i,j}^{n}) n\in1...N_{t}$ (S13)

Where *h_t_* refers to the time step, *N_t_* to the total number of time steps and $\phi{}_{i,j}^{n}$ to substrate concentration in *node* *i,j* and time *n*. The Laplacian of Eq. S13 (∇^2^) is discretised in a two-dimensional space (x, y) using the central finite-difference method (Eq. S14), where *h* is the grid size (*h* = *∆x* = *∆y*).

$\nabla^{2}\phi_{i,j}^{n}=\frac{\phi_{i-1,j}^{n}+\phi_{i+1,j}^{n}+\phi_{i,j-1}^{n}+\phi_{i,j+1}^{n}-4\phi_{i,j}^{n}}{h^{2}}$ (S14)

Eq. S14 (also known as discrete Laplacian) is given as the following kernel (Eq. S15), and the Laplacian approximation is re-written in a matrix from (Eq. S16), where the convolution of $\left[ L \right]$ and $\phi_{i,j}^{n}$ is denoted using the symbol *.

$[L]=\left( \begin{matrix} 0 & 1 & 0 \\ 1 & -4 & 1 \\ 0 & 1 & 0 \end{matrix} \right)$ (S15)

$\nabla^{2}\phi_{i,j}^{n}=\frac{1}{h^{2}}(\left[ L \right]*{[\phi}^{n}])$ (S16)

Where ${[\phi}^{n}]$ is the concentration of soluble compounds ($\phi_{i,j}^{n}$) defined in matrix form (Eq. S17).

${[\phi}^{n}]=\left( \begin{matrix} \begin{matrix} \phi_{1,1}^{n} & \phi_{1,2}^{n} \\ \phi_{2,1}^{n} & \phi_{2,2}^{n} \end{matrix} & \cdots& \begin{matrix} \phi_{1,N_{y}}^{n} \\ \vdots\end{matrix} \\ \begin{matrix} \vdots& \end{matrix} & \ddots& \vdots\\ \begin{matrix} \phi_{Nx,1}^{n} & \cdots\end{matrix} & \cdots& \phi_{N_{x},N_{y}}^{n} \end{matrix} \right)$ $\in M_{N_{x}\times N_{y}}$ (S17)

For discrete and 2-dimensional variables (A and B), Eq. S18 defines the convolution of A and B (i.e., A$*$B). Convolution satisfies the distributive property, multiplicative identity, and associative property with scalar multiplication. These properties are essential to rearrange the diffusion-reaction equation properly.

$A\left[ x \right]*B\left[ x \right]=\sum_{k=-\infty}^{+\infty} A[x]\cdot B[x-k]$ (S18)

Then the diffusion-reaction equation (Eq. S12) can be re-written as a system of matrixes (Eq. S19), where $\psi$ is a constant defined for each soluble component (Eq. S20) and $[I_{k}]$ is the so-called *identity kernel* or *do-nothing convolution kernel* (Eq. S21).

$\left( [I_{k}]-\psi\cdot\left[ L \right] \right)*\left[ \phi^{n+1} \right]= \left( [I_{k}]+\psi\cdot\left[ L \right] \right)*[\phi^{n}]+R(\left[ \phi^{n} \right])\cdot h_{t}$ (S19)

$\psi=\frac{\mathbb{D\cdot}h_{t}}{2\cdot h^{2}}$ (S20)

$[I_{k}]=\left( \begin{matrix} 0 & 0 & 0 \\ 0 & 1 & 0 \\ 0 & 0 & 0 \end{matrix} \right)$ (S21)

## Boundary conditions

To solve Eq. S19 in all the simulation domain, it is necessary to define the boundary conditions of the problem. In this case, a single boundary condition type is defined – the concentrations at outside of the aggregate are defined by the conditions of the bulk liquid. This is implemented by the Dirichlet boundary condition (Eq. S22).

$\phi_{-1,j}^{n}=\phi_{N_{x}+1,j}^{n}=\phi_{i,-1}^{n}= \phi_{i,N_{y}+1}^{n}=\gamma$ (S22)

To implement Dirichlet boundary condition in this system, first the region of the simulation domain is determined. Then, which nodes belong to the diffusion region (comprising boundary layer and granular region) are determined (explained below). Like this, the concentration matrix ${[\phi}^{n}]$ is modified accordingly (Eq. S23): those nodes in which diffusion region is considered have the corresponding concentration value ($[\phi^{n}]$), and that ones in which no-diffusion region is considered have the boundary value (i.e., concentration of bulk liquid, $\gamma$).

$\left[ \phi^{n} \right]^{\gamma}= diffR\circ\left[ \phi^{n} \right]+\mathrm{inv}\left( diffR \right)\cdot\gamma$ (S23)

Where $diffR$ is a logical matrix with 1 (*true*) in those nodes of diffusion region, and 0 (false) in those nodes of no-diffusion region; $\mathrm{inv}\left( diffR \right)$ is the inverse of $diffR$. Then, the diffusion-reaction equation including the boundary conditions imposed over the simulation domain is written in Eq. S24. For each soluble component and time iteration, Eq. S24 is solved calculating $\phi^{n+1}$ using an efficient multigrid method (V-cycle) (6).

$\left( [I_{k}]-\psi\cdot\left[ L \right] \right)*\left[ \phi^{n+1} \right]= \left( \left[ I_{k} \right]+\psi\cdot\left[ L \right] \right)*\left[ \phi^{n} \right]^{\gamma} +R(\left[ \phi^{n} \right])\cdot h_{t}$ (S24)

## Bulk liquid concentrations

In this study its considered that aggregates grow in a continuous stirred tank reactor (CSTR), where substrate concentrations in the bulk liquid are dynamic. The average microbial activity of the aggregate (as a representation of the activity of the whole reactor) is used to integrate the concentration of the soluble components in the bulk liquid of the reactor (S) through a mass balance (Eq. S25).

$\frac{dS}{dt}= \frac{1}{HRT}\cdot\left( S_{inf}-S \right) + R$ (S25)

Where *HRT* refers to the hydraulic time fixed in the reactor, *S_inf,i_* to the concentration in the influent and *R* to the reaction term considered in reactor. As mentioned, the reaction term (*R*) is calculated assuming the average of all the nodes (Eq. S26).

$R = \frac{\sum_{i=1,j=1}^{i =N_{x},j =N_{y}} {R_{i,j}}^{n}}{N_{x}\cdot N_{y}}$ (S26)

The new concentration *S* calculated with the integration of Eq. S25 (using the build-in ode45 solver in MATLAB) updates the Dirichlet value ($\gamma$) included in Eq. S23.

## Domain definition – diffusion and no-diffusion regions

The diffusion region, where diffusion-reaction equation is solved (Eq. S24), comprises the aggregate region and boundary layer. The aggregate region corresponds to cells in which bacteria are growing. Boundary layer is the region of grid cells in the immediate vicinity of boundary layer. The thickness of the boundary layer can be selected by the user.

The distinction of the diffusion region starts with the detection of which nodes are potentially in this region, creating a preliminary diffusion region. For that, the minimum and maximum position of bacteria on aggregate in coordinate *x* and *y*, and boundary layer thickness are considered. The computational cost is reduced by using this preliminary diffusion region instead of the entire simulation domain.

Then, grid cells with bacteria (at least one individual) are determined and included in the diffusion region defining the aggregate region. To recognise which grid cells are belonging to the boundary layer region (and including them in diffusion region), the boundary grid cells with bacteria (i.e., the outermost grid cells of aggregate region) must be sought. An efficient way to find them is through the convolution of aggregate region matrix and *edge detection kernel* (Eq. S27).

$\left[ K \right]_{ED} =\left( \begin{matrix} -\frac{1}{8} & -\frac{1}{8} & -\frac{1}{8} \\ -\frac{1}{8} & 1 & -\frac{1}{8} \\ -\frac{1}{8} & -\frac{1}{8} & -\frac{1}{8} \end{matrix} \right)$ (S27)

Once boundary grid cells with bacteria are found, it is time to check whether the neighbouring grid cells ($g{}_{i,j}$) belong to the boundary layer region and, thus, the diffusion region. By dividing the boundary layer thickness by grid size (*h*), the extent of the neighbouring grid cells with possibility to belong to the boundary layer region is obtained (Fig. A1).


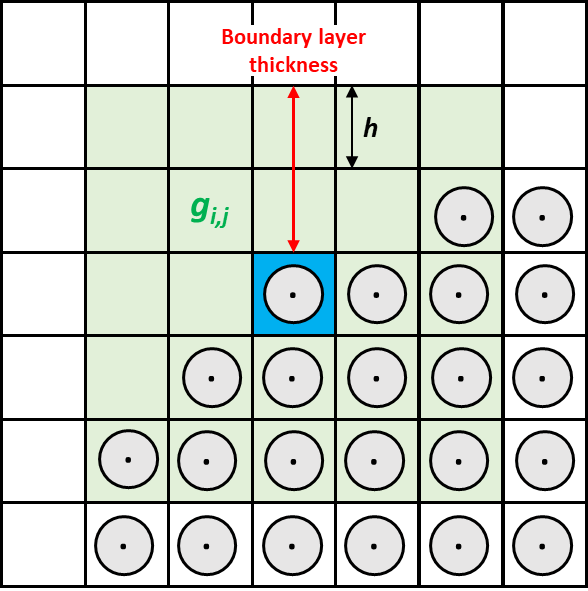


**Fig. A1 Detection of diffusion region.** Neighbouring grid cells (green squares, $g{}_{i,j}$) of a specific boundary grid cell with bacteria (blue square) with potential to be included in diffusion region.

Finally, the focus region is defined, establishing the region of the simulation domain where diffusion-reaction equation is solved, and the pseudo-steady state is checked. The flowchart of the diffusion region determination is presented in Fig. A2.


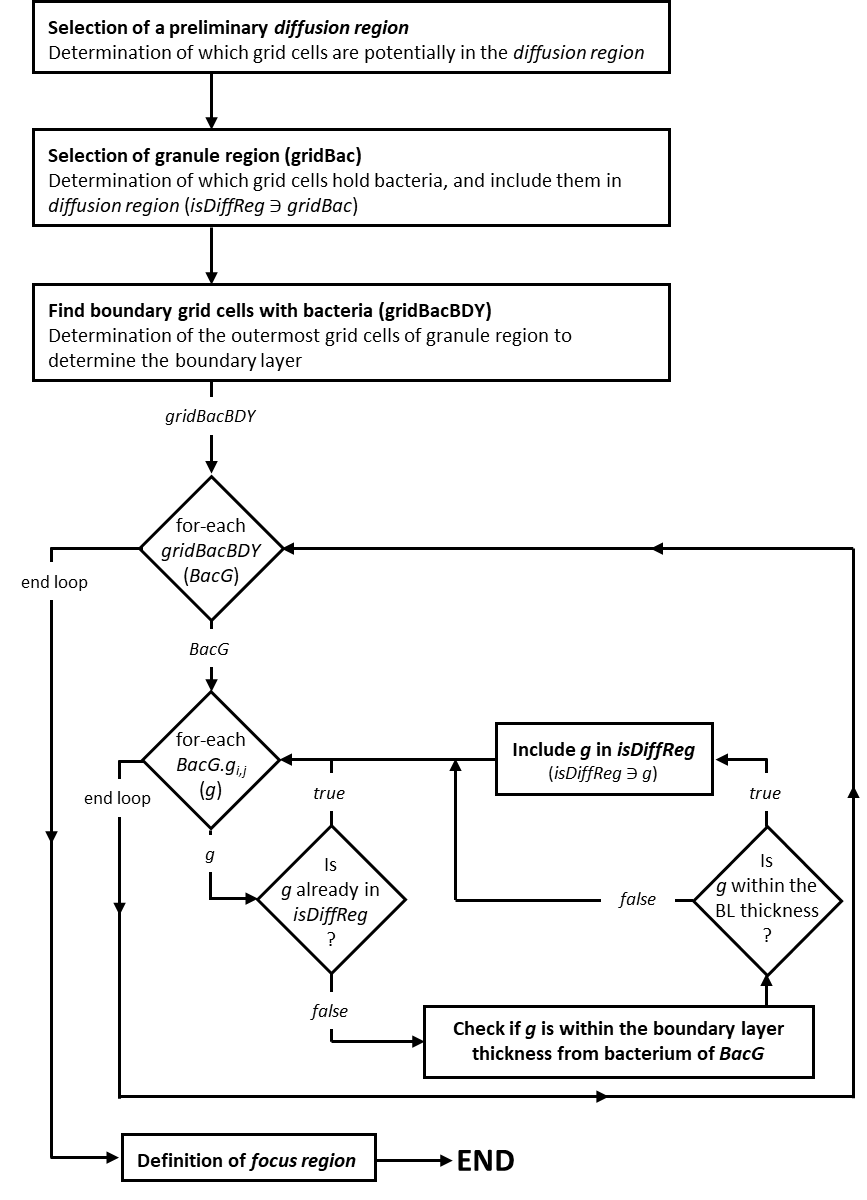


**Fig. A2 Algorithm of diffusion region determination.**

## Reaction term

The matrix of reaction components $R(\left[ \phi^{n} \right])$ must be calculated and added to Eq. S24. The synthesis and consumption of soluble components are assumed to be the result of microbial activity in the aggregate.

The kinetics of the microbial activity are calculated function of the local conditions of the node where the cell is located. The stoichiometry of microbial species is also necessary to compute the synthesis and consumption of soluble components. Eqs. S28 – S30 show examples of stoichiometries for anabolism (*Ana*), catabolism (*Cat*) and decay (*Dec*), where *C_s_* and *N_s_* refer to carbon and nitrogen sources, *eD* and *eA* to electron donor and acceptor and *X* to biomass considering an average formula of C_1_H_1.8_O_0.5_N_0.2_ (7, 8).

$Ana = 1\cdot C_{s}+0.2\cdot N_{s} +\cdots\to\cdots+ 1\cdot X$ (S28)

$Cat = \alpha\cdot eD + \beta\cdot eA \to\cdots$ (S29)

$Dec = 1\cdot X+\cdots\to1\cdot C_{s}+0.2\cdot N_{s}$ (S30)

The stoichiometry of the overall metabolism (*Met*) is calculated function of the catabolic and anabolic stoichiometries, and the growth yield (Y_XS_, Eq. S31).

$Met = \frac{1}{Y_{XS}}\cdot Cat+Ana$ (S31)

With the metabolic stoichiometry and the kinetic parameters (*µ^max^*, *K_S_*, *K_I_* and *b^max^*) for each of the microbial species present in the reactor, the growth rate is calculated (*µ*, Eq. 8; main manuscript). Then, through the stoichiometry and the bacterial mass, the growth/decay of the cell, substrate uptakes and product generations are calculated (Eqs. S32 and S33, respectively). Eq. S32 is the derivative of the mass of a microbe which is integrated in time using a forward Euler scheme; and Eq. S33 computes the reaction term for a specific soluble component *s* due to the activity of the cell *m*.

$\frac{dX_{m}}{dt} = \mu_{m}^{n}\cdot X_{m}^{n}$ (S32)

$R_{m}^{n} = \frac{\mu_{m}^{n}\cdot\delta_{s,m}}{V_{xy}}$ (S33)

Where *X_m_* refers to the mass in moles of the cell *m*, *δ_s,m_* to the stoichiometric coefficient of the substrate S for the cell *m* and *V_xy_* to the volume of one node of the simulation domain. The calculation of the reaction term for the soluble components is function of the position in the simulation domain (one reaction term per node for each soluble component). Eq. S34 is evaluated considering all the microorganisms that are contributing to the reaction term of a specific soluble component in the *node i,j*.

$R_{i,j}^{n}=\sum_{m = 1}^{m = M_{i,j}} R_{m}^{n}$ (S34)

Where *M_i,j_* refers to all microorganism that are in the *node i,j.* The reaction terms *R_ij_^n^* calculated by Eq. S34 for all nodes of the simulation domain ($R(\left[ \phi^{n} \right])$), are then used in Eq. S24 to calculate the concentration of each of the soluble components in the simulation domain.

## Shoving algorithm

To compute the shoving of the cells in the aggregate after cell growth and division, first the overlap between microorganisms is checked by Eq. S36.

$\left| \vec{v} \right|=\sqrt{\left( x_{m}-x_{m+1} \right)^{2}+\left( y_{m}-y_{m+1} \right)^{2}}$ (S35)

${overlap=kDist\cdot(r}_{m}+r_{m+1}) -\left| \vec{v} \right|$ (S36)

Where |$\vec{v}$| is the norm of the vector that links the centres of both cells *m* and *m+1* and $kDist$ is just a multiplier that allows adjustment of the minimal spacing between bacteria. In this case, the *quadtree algorithm* is applied to detect the *overlapping* between bacteria. Subsequently, the shoving of these bacteria that overlap is computed (9). If the *overlap* value is bigger than the distance allowed by the user, then microorganisms are pushing each other function their mass and distance (Eqs. S37 – S42).

$\vec{p}=\frac{kDist\cdot{(r}_{m}+r_{m+1}) -\left| \vec{v} \right|}{\left| \vec{v} \right|}$ (S37)

$a_{m}=1-\frac{X_{m}}{X_{m}+X_{m+1}} ; a_{m +1}=1-\frac{X_{m+1}}{X_{m}+X_{m+1}}$ (S38)

$x_{new, m}= x_{old}-\left( x_{m+1}-x_{m} \right)\cdot a_{m}\cdot\vec{p}$ (S39)

$y_{new,m}= y_{old}-\left( y_{m+1}-y_{m} \right)\cdot a_{m}\cdot\vec{p}$ (S40)

$x_{new, m+1}= x_{old}+\left( x_{m+1}-x_{m} \right)\cdot a_{m +1}\cdot\vec{p}$ (S41)

$y_{new,m+1}= y_{old}+\left( y_{m+1}-y_{m} \right)\cdot a_{m +1}\cdot\vec{p}$ (S42)

Overlap assessment and shoving estimation (if needed) are only performed over the neighbour individuals. Because bacteria are not motile and therefore will not move very far from the original position, it is assumed that chosen neighbouring bacteria stay constant. An overview of the shoving algorithm is presented in Fig. A3.

**
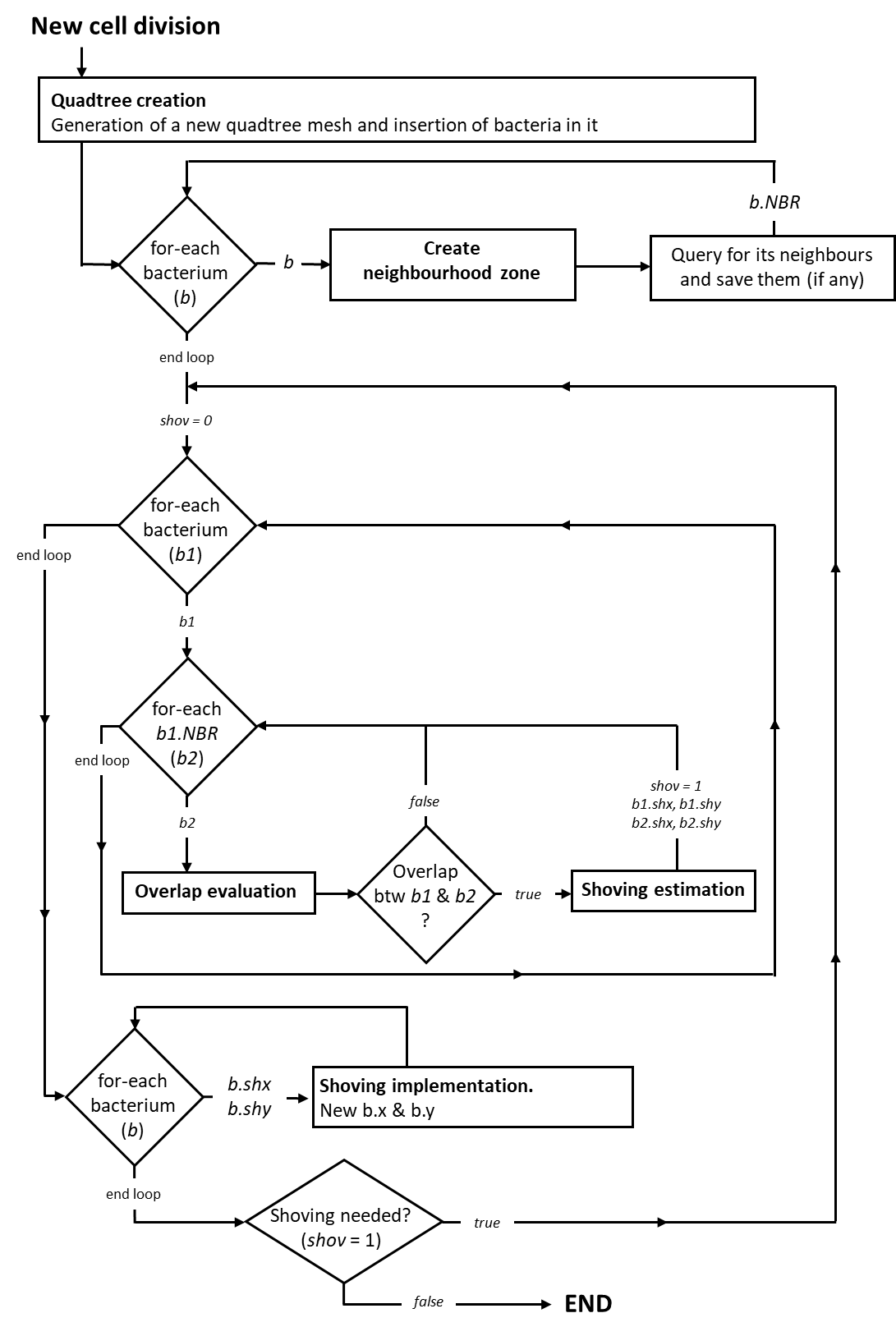
**

**Fig. A3 Scheme of shoving algorithm.** Legend: *b.shx* and *b.shy* – displacement of bacterium *b* due to shoving of the others; *b.NBR* – neighbours of bacterium *b*.

## Integration

Cells are dividing in a time scale much slower than the diffusion-reaction process (~1 hour versus ~10^‑8^ hours). To solve the system, the model takes advantage of this time scale differentiation to separate the processes of solving the diffusion-reaction equation, and the cell division and its shoving (10).

First, the diffusion‑reaction equation is integrated (with a time step *dt*) until it reaches a pseudo‑steady state, allowing a deviation of up to 1% from the real steady state (Eqs. S43 and S44). To check whether pseudo-steady state is reached or not, only diffusion region is considered, as the Dirichlet boundary condition and the bulk liquid concentrations are constants in this specific time span, changing only when the microbial community change significantly (due to a cell division, cell inactivation or a substantial variation in microbial mass).

$\left[ RES \right]=\left[ L \right]*\left[ \phi\right]^{\gamma}+\left( {h^{2}}/\mathbb{D} \right)\cdot R\left( \left[ \phi\right] \right)$ (S43)

$Tol\boldsymbol{:=}max\left| \frac{RES_{i,j}}{1\cdot10^{-4}+\phi_{i,j}} \right|\leq1\%, Tol:=\left\{ \begin{aligned} max\left| RES_{i,j} \right|\leq1\%\cdot\phi_{i,j}, if \phi_{i,j}\gg1\cdot{10}^{-6} \\ max \left| RES_{i,j} \right|\leq1\cdot{10}^{-6}, if \phi_{i,j}\ll1\cdot{10}^{-6} \end{aligned} \right.$ (S44)

When the pseudo-steady state is reached, the biomass growth is integrated in a bigger time step (*dt_bac_*). Once the mass of all individuals is updated, the mass balances of the overall reactor are integrated with the the time step *dt_bac_* considering the average microbial activity of the aggregate. After that, the Dirichlet boundary condition and the reaction term are updated. At the end of this bigger step, the diffusion-reaction equation needs to be integrated again to reach a new pseudo-steady state.

An overall scheme of model integration, and summary of parameters are presented in Fig. A4 and Table S2, respectively.

**
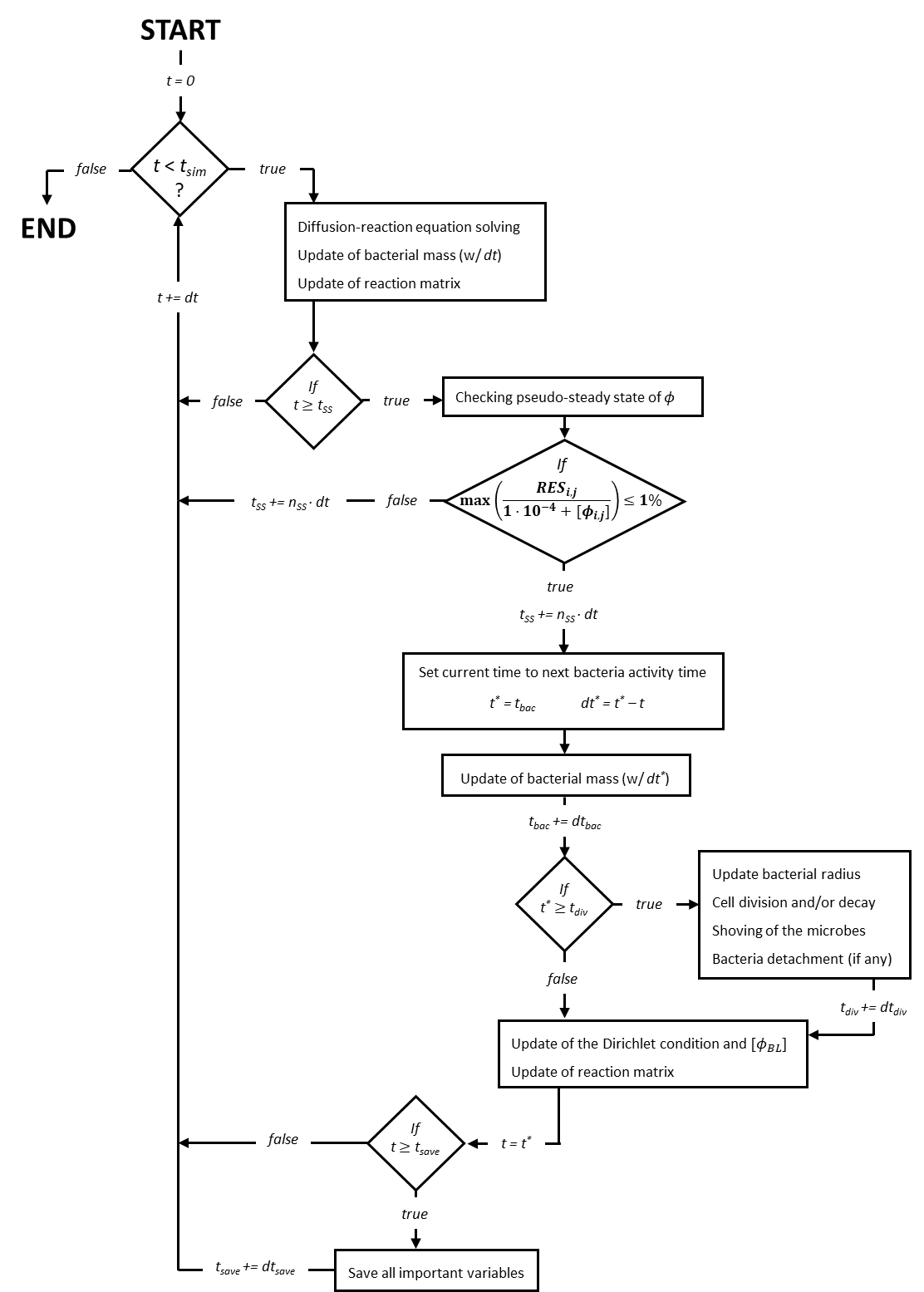
**

**Fig. A4 Algorithm scheme of the integration process.**

## Summary of model boundary, range of application and model assumptions

**· Model boundary and range of application.** The aim of this study is to understand the mechanistic dynamics behind the two early studies of comammox *Nitrospira* (11, 12), evaluating its resilience under distinct limiting environments and its potential to establish a stable community with anammox bacteria. Daims *et al.* (11) and van Kessel *et al.* (12) observed the formation of flocs in their experiments. In order to model these flocs as accurately as possible, the simulation domain is initialized with multiple small microbial aggregates. The range of application of the model presented here are the early studies of comammox *Nitrospira*, where specific community (*Nitrospira* and anammox bacteria) and environmental conditions (feeding regime and oxygen concentration) were selected.

This model is capable to simulate any other microbial community in self-aggregated systems: suspended flocs (this study), or denser aggregates/granules (13). For more information, see the public GitHub repository at [Computational-Platform-IbM/IbM (github.com)](https://github.com/Computational-Platform-IbM/IbM).

**· Model assumptions.** Assumptions are the set of explicitly stated (or implicit premised) conventions and choices that fulfil the holes in our interpretation of reality. These establish the limits of our model and simplify the problem. Main assumptions applied in this model are listed below:

- Cell division and inactivation occur at a certain microbe’s size, independently of the state of other cells. Cell growth and decay is established by the actual specific growth rate (*µ*): *µ* > 0 – microbial growth; *µ* < 0 – microbial decay.
- No lysis cellular is considered. When a cell reach a minimum size, this becomes inactive.
- Only diffusive transport of substrate and products throughout boundary layer and aggregate regions is considered. Convection transport of substances is not considered here.
- The growth of each individual is only affected by local conditions, which in turn are influenced by the activity of surrounding microbes.
- The system was simulated as an ideal continuous stirred tank reactor.
- Simulated aggregates are considered a representation of the activity of the whole reactor.
- Bulk liquid dynamics is only influenced by the microbial activity in aggregates except oxygen, which is assumed to be constant in bulk liquid. Planktonic cells are not considered in this study.
- Non-kinetic competition: the kinetic parameters defining the Monod curve for the different populations (*µ_max_*, K_N_, K_O2_, $a$) are assumed equal for *Nitrospira* and anammox bacteria.
- The distinct metabolic efficiencies of comammox *Nitrospira* (growth yield, Y_X/S_) are estimated based on thermodynamics, first principles and biochemistry of comammox *Nitrospira*.
- Static metabolism of *Nitrospira* is assumed. Metabolism of *Nitrospira* cells is set at the beginning of the simulation and remains throughout the simulation time.
- The presence of certain capacity for oxygen protection of aerobic *Nitrospira* to anammox bacteria is ensured assuming ${K_{I,O_{2}}^{AMX}}/{K_{O_{2}}^{Ns}}=1$.
- Active movement (cell motility), dispersion/invasion, nor loss of microorganisms with effluent is considered. Microbes can move passively due to shoving forces exerted by neighbouring cells as they grow and divide.

# Supplementary Results/Discussion – (Eco)physiological analysis of comammox *Nitrospira*

Currently, physiological characterizations of *Nitrospira inopinata* (14) and *Candidatus* Nitrospira kreftii (15) are available. In these studies, it was reported that although both comammox species have similar ammonia affinity (0.063 µM NH_3_ for *N. inopinata*, and 0.040 µM NH­_3_ for *Ca.* N. kreftii), they have different nitrite affinities (449.2 µM NO_2_^-^ for *N. inopinata*, and 12.5 µM NO_2_^-^ for *Ca.* N. kreftii). Nitrite affinity of comammox *Nitrospira* might play a fundamental role in microbial community assembly (especially on competition between NO, An-NRMX and AMX for nitrite) and transient accumulation of nitrite. To evaluate this, the following simulation experiments were carried out:

- Non-equimolar feeding regime (NH_3_:NO_2_^-^:NO_3_^-^ = 500:375:500 µM) and hypoxia conditions (1.0 µM, 1.5 µM and 3.0 µM of O­_2_) assuming nitrite affinity of comammox *Nitrospira* equal to 12.5 µM NO_2_^-^ and 449.2 µM NO_2_^-^ (Fig. S8).
- Only ammonia feeding (1000 µM NH_3_) and aerobic conditions (93.8 µM O_2_) assuming nitrite affinity of comammox *Nitrospira* equal to 12.5 µM NO_2_^-^ and 449.2 µM NO_2_^-^ (Fig. S9).

The suppression of NO and An-NRMX activities was higher when reducing the nitrite affinity of comammox *Nitrospira* (i.e., increasing the value of K_NO2_). NO activity was also fully suppressed at 1.5 µM of O_2_, whereas An-NRMX was no longer active at ≤ 1.5 µM of O_2_ (Figs. S8B and S8C). When nitrite affinity was assumed equal to 449.2 µM of NO_2_^-^, NO activity consisted only 2.9 ± 3.5 wt. % of the whole community at 3.0 µM of O_2_ (Fig. S8C). A smaller nitrite affinity (higher K_NO2_ value) meant a reduction of competitive capacity of both activities (NO and An-NRMX) to survive against the stronger competitor (AMX).

Intriguingly, AMX remained active at 3.0 µM of O_2_ after reducing the nitrite affinity of comammox *Nitrospira* to 12.5 µM NO_2_^-^ (Fig. S8B) and 449.2 µM NO_2_^-^ (Fig. S8C), while AMX was not active at 3.0 µM of O_2_ assuming a nitrite affinity of comammox *Nitrospira* equal to 1.0 µM of NO_2_^-^ (Fig. S8A). Additionally, higher proportion of AMX at 3.0 µM of O_2_ was observed as lower nitrite affinity of comammox *Nitrospira* was assumed (see labels over bars of Fig. S8). This is also associated to the reduction of competitive capacity of NO activity, suggesting that the suppression of AMX at the main simulation setup (i.e., K_NO2_ value of comammox *Nitrospira* equal to 1.0 µM of NO_2_^‑^; Fig. S8A) was a combination of oxygen inhibition and the presence of a competitive NO activity. Like in the main simulation setup under 1.0 µM and 1.5 µM of O_2_ (bottom panel of Fig. S8A), no stratification of aerobic comammox *Nitrospira* (performing AO, NO, CMX or NRMX) and anammox bacteria was observed (bottom panels of Figs. S8B and S8C).

Regarding transient accumulation of nitrite, a significant increase of nitrite accumulation was only observed when nitrite affinity of comammox *Nitrospira* was set to 449.2 µM NO_2_^-^ (*p* < 0.002; Fig. S9), being the peak of nitrite close to the levels observed experimentally by Daims *et al.* (2015) (30% of the added ammonia) (ref. 11).

The results presented here suggest that the taxonomy of comammox *Nitrospira* would have a significant impact on the co-existence of anammox bacteria and comammox *Nitrospira*. In this case, *N. inopinata*, with a lower nitrite affinity, would be a better partner of anammox bacteria than *Ca.* N. kreftii. In fact, the first stable partnership between anammox bacteria and comammox *Nitrospira* was achieved with *N. inopinata* (16).

# Supplementary Figures

| **A)**  **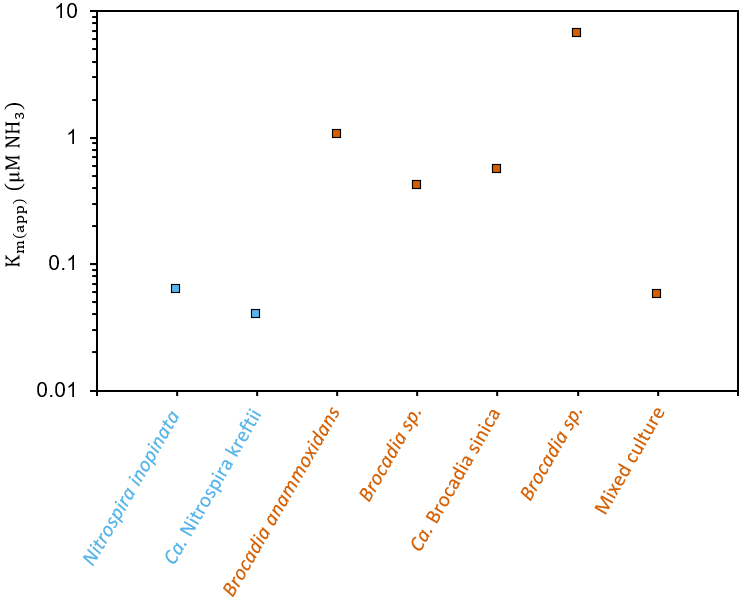** |
| --- |
| **B)**  **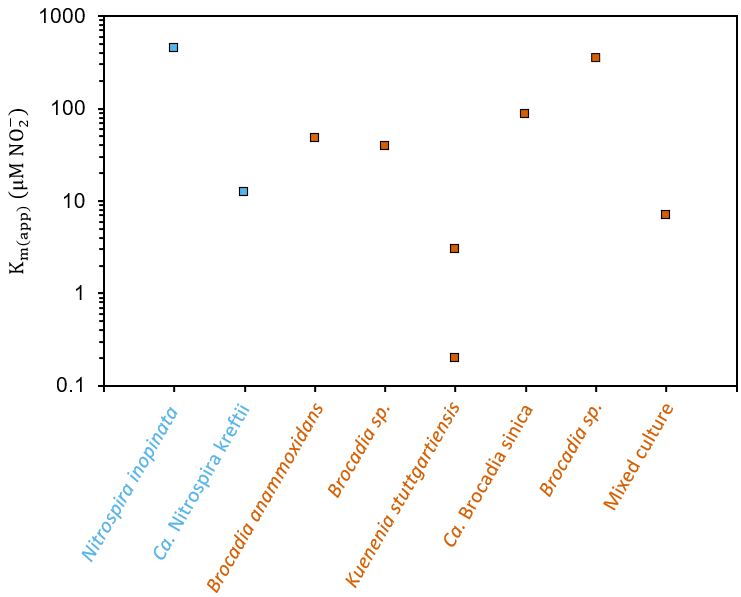** |

**Fig. S1 Apparent substrate affinity (K_m(app)_) for ammonia (panel A) and nitrite (panel B) of comammox *Nitrospira* and anammox bacteria.** Colour legend: light blue – comammox *Nitrospira*; orange – anammox bacteria. References of substrate affinities for: comammox *Nitrospira* – (14, 15); anammox bacteria – (17-22).

|  | **A) Ammonia feeding** | **B)  Equimolar feeding** | **C) Non-equimolar feeding** |
| --- | --- | --- | --- |
| **1.0 µM O_2_** | **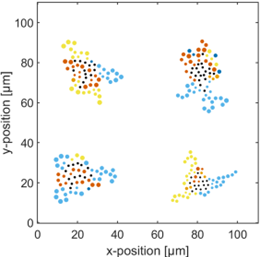** | **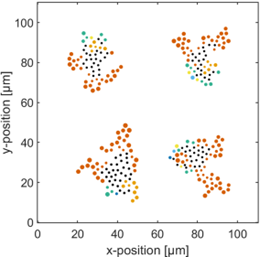** | **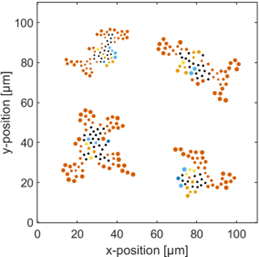** |
| **1.5 µM O_2_** | **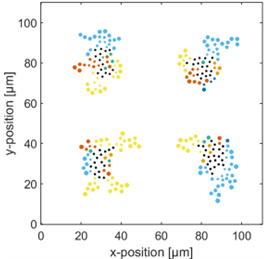** | **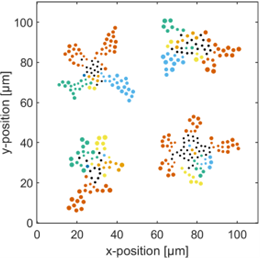** | **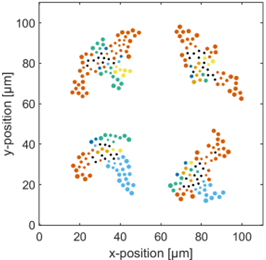** |
| **3.0 µM O_2_** | **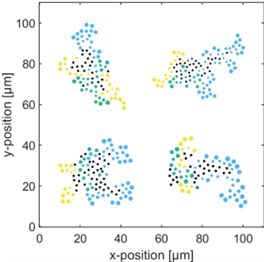** | **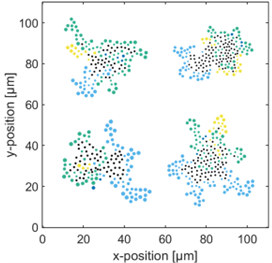** | **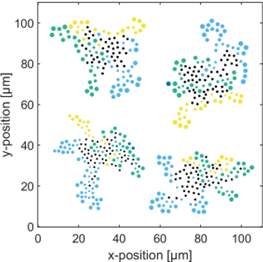** |
| **93.8 µM O_2_** | **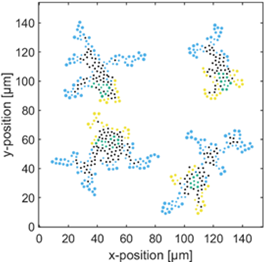** | **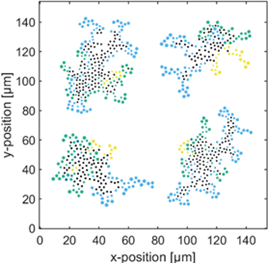** | **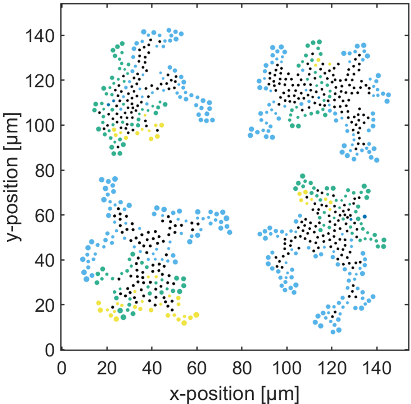** |
|  | AO  NO  CMX  NRMX  An-NRMX  AMX | | |

**Fig. S2 Additional floc images of comammox *Nitrospira* and anammox bacteria community from simulations at different nitrogen feeding regimes and oxygen concentrations (1.0, 1.5, 3.0, 93.8 µM).** Ammonia feeding (NH_3_:NO_2_:NO_3_ = 500:0:0 µM). **B)** Equimolar feeding (NH_3_:NO_2_:NO_3_ = 500:500:500 µM). **C)** Non-equimolar feeding (NH_3_:NO_2_:NO_3_ = 500:375:500 µM). Black circles represent inactive individuals.

| **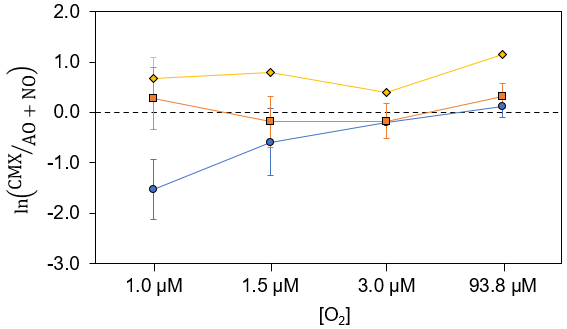**  **A)** | **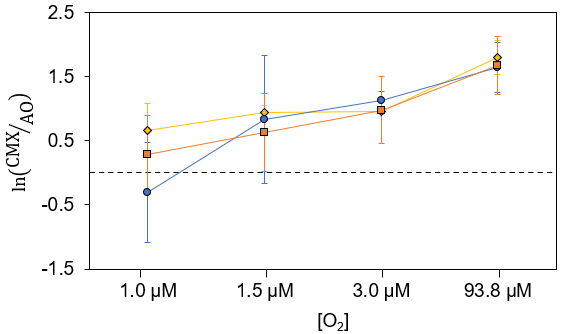**  **B)** |
| --- | --- |
| \| **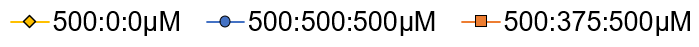** Ammonia feeding  **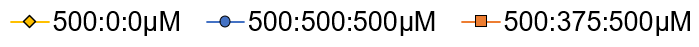** Equimolar feeding **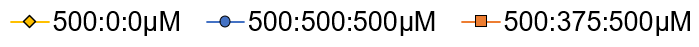** Non-equimolar feeding \| \| --- \| | |

**Fig. S3 Metabolic ratios of *Nitrospira* (AO, NO and CMX) represented as ln(A/B) at different nitrogen feeding regimes (NH_3_:NO_2_:NO_3_ ratio) and oxygen concentrations.** **A)** Metabolic ratios between CMX and division of labour (AO+NO). **B)** Metabolic ratios between CMX and AO. Error bars show standard deviation of *n* = 3 simulation replicates. Feeding regimes – ammonia feeding: 500:0:0 µM; equimolar feeding: 500:500:500 µM; non-equimolar feeding: 500:375:500 µM. The statistical significance between the different nitrogen feeding regimes is shown in Table S9.

**
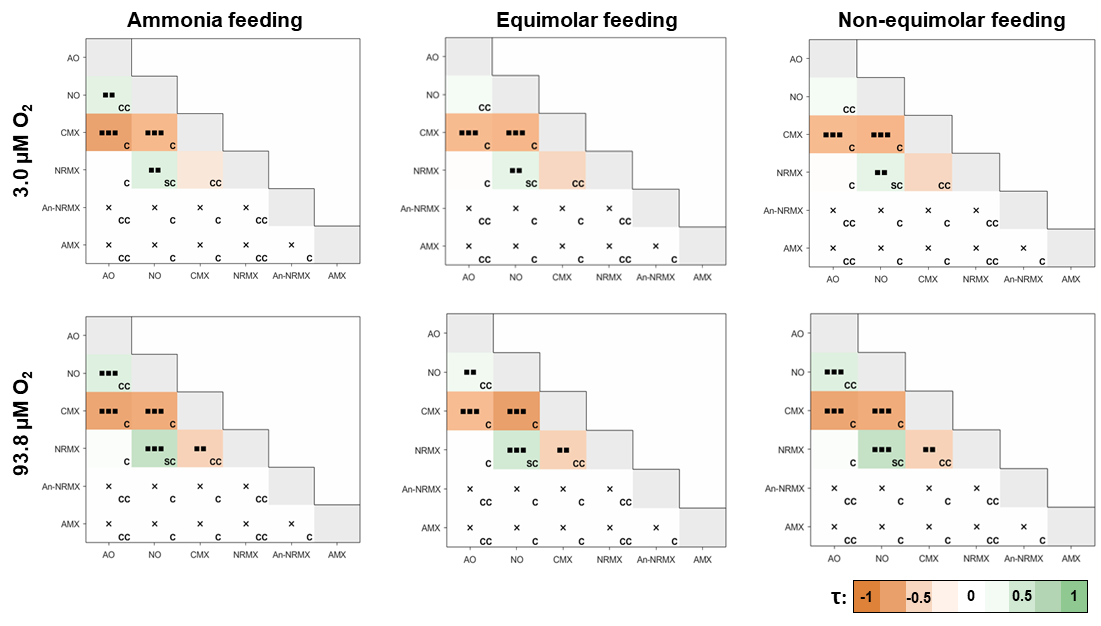
**
**Fig. S4 Ecological analysis at floc level under conditions where only comammox *Nitrospira* remained active (3.0 and 93.8 µM of O_2_).** Ammonia feeding (NH_3_:NO_2_:NO_3_ = 500:0:0 µM, left panels), equimolar feeding (NH_3_:NO_2_:NO_3_ = 500:500:500 µM, centre panels), and Non-equimolar feeding (NH_3_:NO_2_:NO_3_ = 500:375:500 µM, right panels). The correlation coefficients of metabolisms are presented on a colour scale. Dotted cells indicate statistically significant correlation (▪ : *p* < 0.05; ▪▪ : *p* < 0.01; ▪▪▪ : *p* < 0.001). Cross symbol (x) indicates no co-existence of the metabolic pair at the end of the simulation experiments. Bottom-right labels indicate the ecological interaction of metabolic pair: CC – Commensalism + Competition; SC – Syntrophism + Competition; C – Competition. Sample sizes employed for Kendall’s τ calculation are shown in Fig. S5.

**
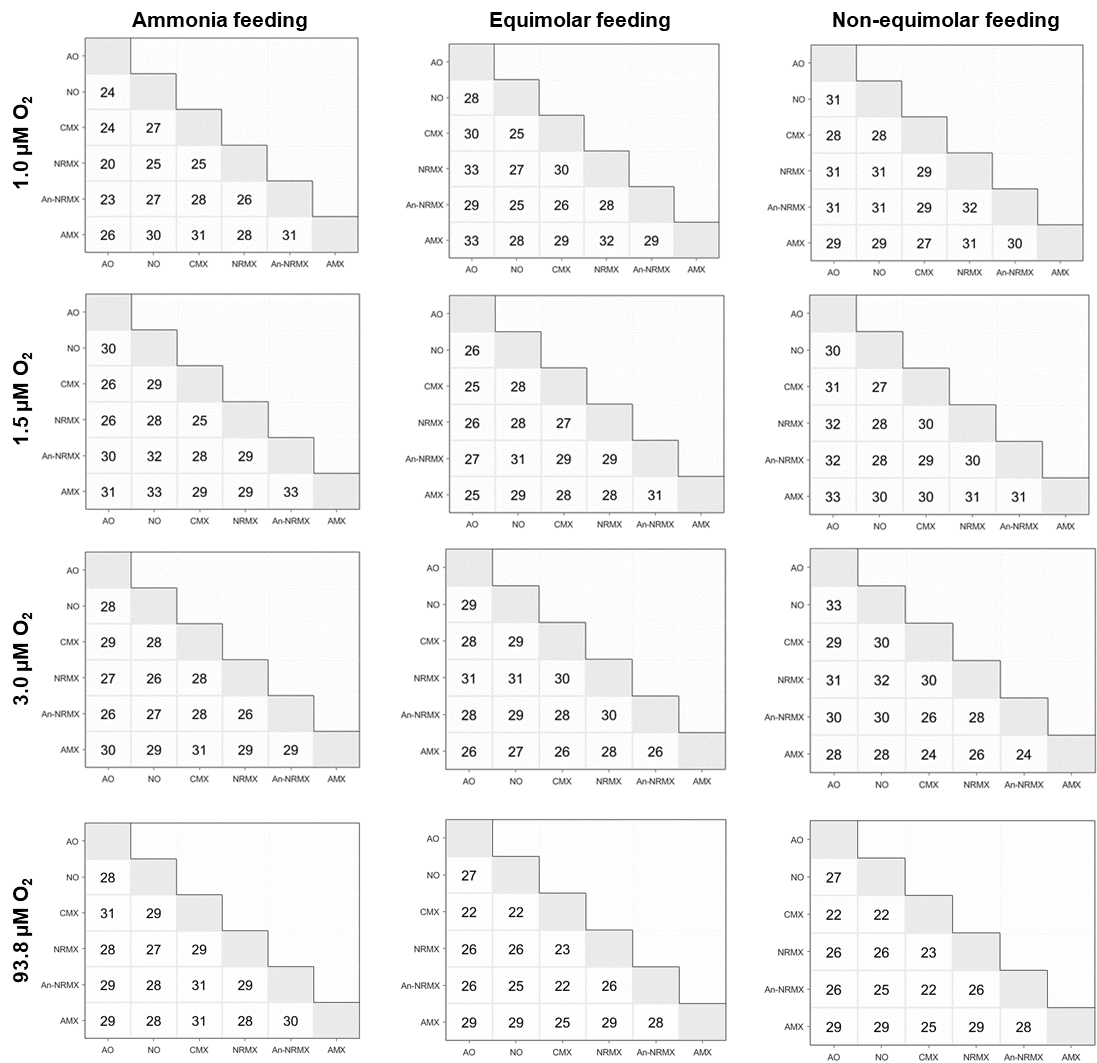
**

**Fig. S5 Sample sizes employed for Kendall’s coefficient calculation.** Different nitrogen feeding regime was applied: ammonia feeding (NH_3_:NO_2_:NO_3_ = 500:0:0 µM, left panels), equimolar feeding (NH_3_:NO_2_:NO_3_ = 500:500:500 µM, centre panels), and non-equimolar feeding (NH_3_:NO_2_:NO_3_ = 500:375:500 µM, right panels). Total replicates: 36 in all conditions.


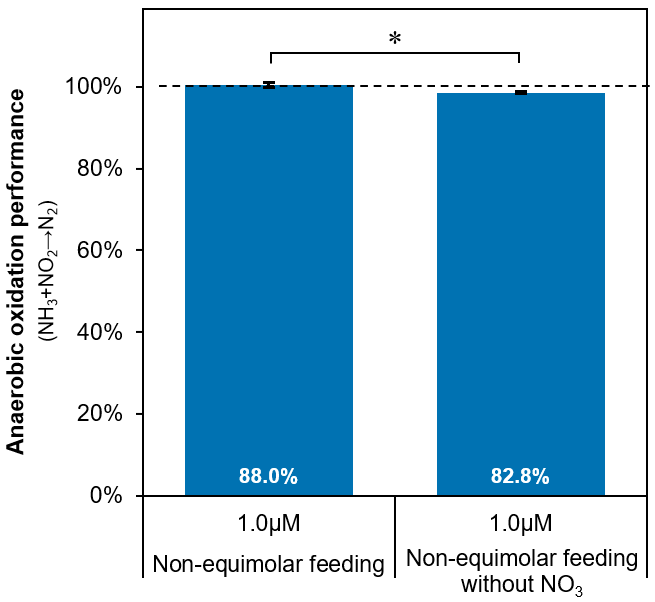


**Fig. S6 Influence of nitrate concentration in the influent for anaerobic oxidation performance (ammonia and nitrite oxidation to N_2_) expressed as percentage.** Data labels depict the relative abundance of anaerobic activities (An-NRMX and AMX). Feeding regimes: non-equimolar feeding – 500:375:500 µM; non-equimolar feeding without NO_3_ – 500:375:0 µM. Error bars show standard deviation of *n* = 3 simulation replicates. Asterisks denote p-value significance where *, p < 0.05. For more information about the calculus of the anaerobic oxidation performance (see Methods – *Parameters for the quantification of nitrogen removal*).


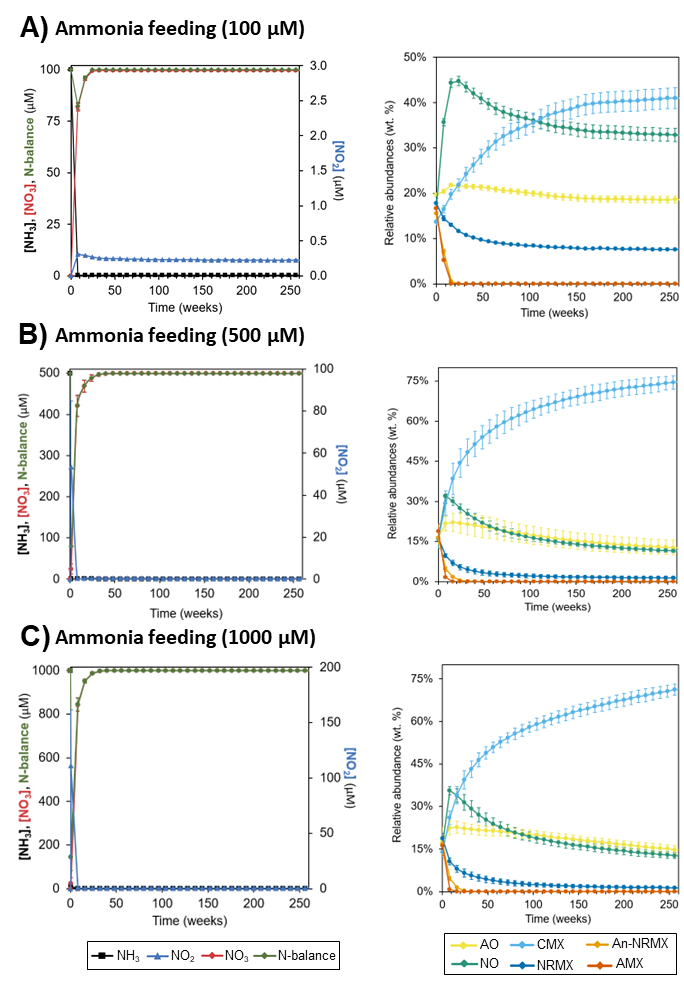


**Fig. S7 Influence of ammonia concentration in the transient accumulation of nitrite at 93.8 µM of O_2_.** Three ammonia concentrations were tested. Full simulation (5.0 years): 100 µM of NH_3_ (panels **A**), 500 µM of NH_3_ (panels **B**), and 1000 µM of NH_3_ (panels **C**). Left panels show the dynamics of nitrogen compounds (NH_3_, NO_2_ and NO_3_) at the early stages of simulation. Right panels show the evolution of relative abundances of comammox *Nitrospira* and anammox bacteria. Error bars show standard deviation of *n* = 3 simulation replicates. If not visible, error bars are smaller than symbols.


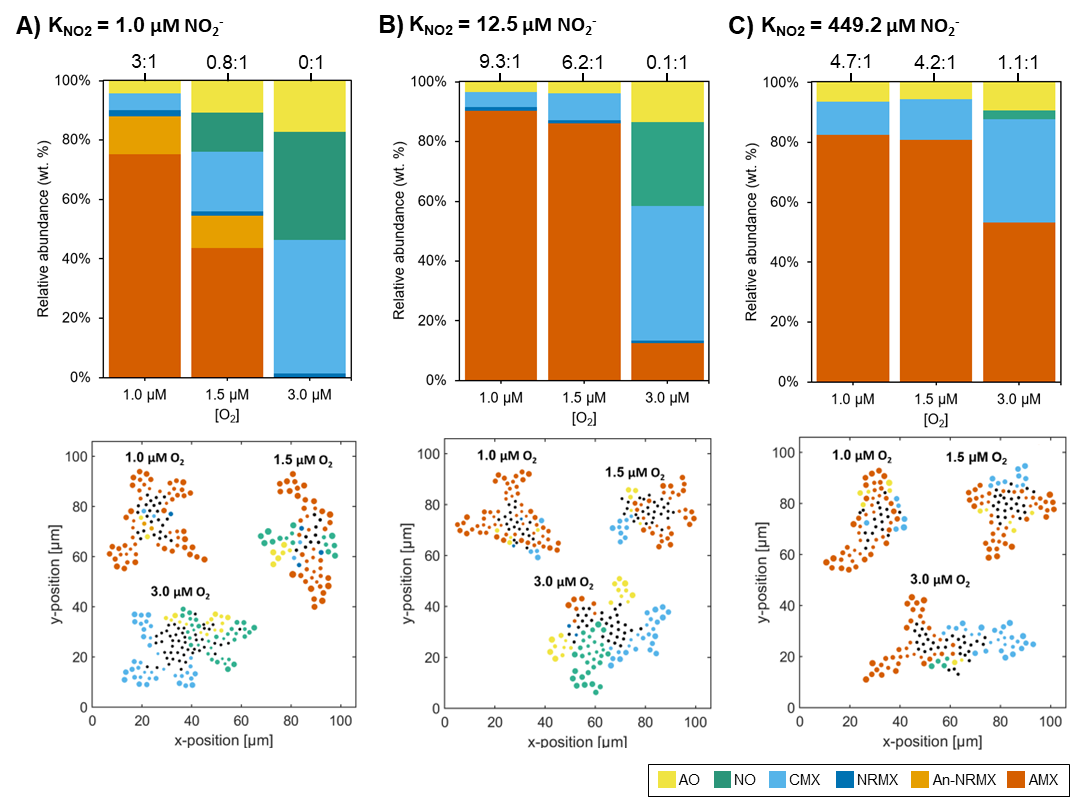


**Fig. S8 Influence of nitrite affinity (K_NO2_) of comammox *Nitrospira* on community assembly applying non-equimolar feeding (NH_3_:NO_2_^-^:NO_3_^-^ = 500:375:500 µM) and hypoxia conditions (1.0 µM, 1.5 µM, 3.0 µM of O­_2_).** Relative abundances of *Nitrospira* metabolic activities (AO, NO, CMX, NRMX and An-NRMX) and anammox bacteria (AMX), with each correspondent floc images (bottom panels) assuming nitrite affinity equal to **A)** 1.0 µM of NO_2_^-^, **B)** 12.5 µM of NO_2_^-^ and **C)** 449.2 µM of NO_2_^-^. Labels over each bar show Anammox:*Nitrospira* ratio at steady state. The statistical significance between different oxygen concentrations and nitrite affinities are shown in Tables S10 and S11, respectively. Black circles on floc images represent inactive individuals.


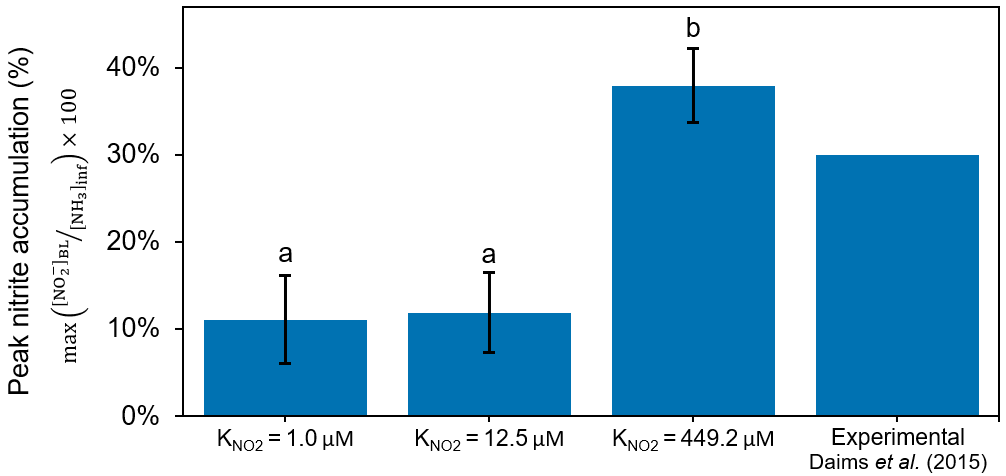


**Fig. S9 Influence of nitrite affinity (K_NO2_) of comammox *Nitrospira* on transient accumulation of nitrite (expressed as percentage) at 93.8 µM O_2_.** Only ammonia was fed (1000 µM NH_3_). Error bars show standard deviation of n = 3 simulation replicates. Bars that do not share similar letters denote statistical significance, *p* < 0.05.

# Supplementary Tables

**Table S1. Key parameter values to calculate** $\boldsymbol{\Delta}\boldsymbol{G}_{\boldsymbol{Cat}}^{\boldsymbol{01}}$ **of *Nitrospira* metabolic activities.** Values of reduction potential at pH 7 ($Ѱ_{i}$) are from eQuilibrator 3.0 (ref. 23). $\gamma_{A}^{*}$ is the number of electrons “accepted” per mole of electron acceptor.

| **Electron acceptor pair** | **Metabolisms** | $\boldsymbol{\gamma}_{\boldsymbol{A}}^{\boldsymbol{*}}$ **(e^-^/mole of eA)** | $\boldsymbol{Ѱ}_{\boldsymbol{i}}$ **(mV)** |
| --- | --- | --- | --- |
| UQ/UQH_2_ | AO, NRMX, An-NRMX | – | 113 |
| O_2_/H_2_O | AO, NO, CMX | 4 | 854 |
| NO_3_/NO_2_ | NO (as eD), NRMX | 2 | 415 |
| NO_2_/NO | An-NRMX | 1 | 385 |

**Table S2. List of general parameters used in all simulation experiments**

| **Parameter** | **Symbol** | **Value** | **Unit** | **REF** |
| --- | --- | --- | --- | --- |
| *Simulation domain* | | | | |
| Domain size | $L_{x}\times L_{y}$ | $514\times514$ | µm |  |
| Cartesian grid cells | $N_{x}\times N_{y}$ | $257\times257$ | grid cells |  |
| Grid cell size | $\Delta x\times\Delta y$ | $2\times2$ | µm |  |
| Initial number of flocs | $n_{floc,init}$ | $12$ | flocs |  |
| Size of initial aggregates | $m_{init}$ | $12$ | individuals |  |
| *Physical parameters: diffusion* | | | | |
| Ammonia (NH_3_) | $D_{NH_{3}}$ | $7.05\cdot{10}^{-6}$ | m^2^ h^-1^ | (ref. 24) |
| Nitrite (NO_2_) | $D_{NO_{2}^{-}}$ | $6.88\cdot{10}^{-6}$ | m^2^ h^-1^ | (ref. 24) |
| Nitrate (NO_3_) | $D_{NO_{3}^{-}}$ | $6.85\cdot{10}^{-6}$ | m^2^ h^-1^ | (ref. 24) |
| Oxygen (O_2_) | $D_{O_{2}}$ | $7.56\cdot{10}^{-6}$ | m^2^ h^-1^ | (ref. 24) |
| Carbon dioxide (CO_2_) | $D_{{CO}_{2}}$ | $6.91\cdot{10}^{-6}$ | m^2^ h^-1^ | (ref. 24) |
| Diffusion correction term | $\sigma_{Diff}$ | $0.7$ | – | (ref. 25) |
| *Physical parameters of bacteria* | | | | |
| Density of biomass | $\rho_{b}$ | $500$ | kg m^-3^ | (ref. 26, 27) |
| Division radius | $r_{max}$ | $1$ | µm | (ref. 28) |
| Inactivation radius | $r_{max}$ | $0.464$ | µm | (ref. 28) |
| Molecular weight (CH_1.8_O_0.5_N_0.2_) | $MW_{b}$ | 24.6 | g mol^-1^ | (ref. 29) |
| Overlap distance coefficient | $kDist$ | $1.5$ | – |  |
| *Reactor parameters* | | | | |
| Boundary layer thickness | $L_{b}$ | 5 | µm | (ref. 30) |
| Representative volume | $V_{r}$ | $1.38\cdot{10}^{-3}$ | m^3^ | (ref. 12) |
| Bulk concentration pH | $pH$ | $7.0$ | – | (ref.12) |
| Temperature | $T$ | $20$ | ^o^C | (ref. 12) |
| Hydraulic retention time | $HRT$ | $140$^(1)^ | h | (ref. 12) |
| *Computational parameters* | | | | |
| Steady-state tolerance | $\mathrm{to}l_{\mathrm{SS}}$ | $0.005$ | mol L^-1^ h^-1^ |  |
| Diffusion tolerance | $\mathrm{to}l_{\mathrm{Diff}}$ | $1\cdot{10}^{-8}$ | mol m^-3^ |  |
| Maximum bulk concentration change | $\Delta\phi_{bulk, max}$ | $2.0$ | % |  |
| ^(1)^Hydraulic retention time calculated based on the experimental setup of van Kessel *et al.* (2015) – reactor size of 7L, cycle times of 12 h and a cycle feed of 600 mL (ref. 12). | | | | |

**Table S3. Summary of oxygen tolerance of anammox bacteria from literature.**

| **Value (µM)** | **REF** | **Comments** |
| --- | --- | --- |
| **Upper oxygen limit (UP)** | | |
| 0.63 | (ref. 31) | Anammox activity measured based on ammonium consumption. |
| 1.00 | (ref. 32) | Anammox activity measured based on ammonium consumption. |
| 3.75 | (ref. 33, 34) | Anammox activity measured as ^15^N^14^N production in ^15^NO_2_- (+^14^NH_4_^+^) and ^15^NH_4_^+^+^14^NO_2_^-^ incubations. From sea. |
| 13.50 | (ref. 35) | Anammox activity measured as ^15^N^14^N production in ^15^NO_2_- (+^14^NH_4_^+^) and ^15^NH_4_^+^+^14^NO_2_^-^ incubations. (Sea) |
| 20.00 | (ref. 36) | Anammox activity measured as ^15^N^14^N production in ^15^NO_2_- (+^14^NH_4_^+^) and ^15^NH_4_^+^+^14^NO_2_^-^ incubations. From sea. |
| 31.25 | (ref. 37) | Anammox activity measured based on ammonium consumption. (Biofilm) |
| 63.00 | (ref. 20) | Anammox activity measured based on ammonium consumption. Aggregate biomass was dominated by anammox bacteria (i.e., scavengers of DO was less than 0.1 %) |
| **Half-maximal activity inhibitory concentration (IC_50_)** | | |
| 0.89 | (ref. 38) | Anammox activity measured as ^15^N^14^N production in ^15^NO_2_- (+^14^NH_4_^+^) and ^15^NH_4_^+^+^14^NO_2_^-^ incubations. From sea. |
| 3.40 | (ref. 36) | Anammox activity measured as ^15^N^14^N production in ^15^NO_2_- (+^14^NH_4_^+^) and ^15^NH_4_^+^+^14^NO_2_^-^ incubations. From sea. |
| 8.00 | (ref. 35) | Anammox activity measured as ^15^N^14^N production in ^15^NO_2_- (+^14^NH_4_^+^) and ^15^NH_4_^+^+^14^NO_2_^-^ incubations. From sea. |
| 11.10 | (ref. 36) | Anammox activity measured as ^15^N^14^N production in ^15^NO_2_- (+^14^NH_4_^+^) and ^15^NH_4_^+^+^14^NO_2_^-^ incubations. From sea. |
| 71.88 | (ref. 39) | Anammox activity measured based on the N_2_ production rate. Suspended Anammox enrichment culture. |
| 118.75 | (ref. 39) | Anammox activity measured based on the N_2_ production rate. Granular Anammox enrichment. |
| **Non-competitive oxygen inhibition constant (K_I,O2_)** | | |
| 0.313 | (ref. 32) | Anammox activity measured based on ammonium consumption. |
| 0.092 | (ref. 22) | Anammox activity measured based on ammonia and nitrite consumption. |

**Table S4. Summary of growth kinetics of comammox *Nitrospira* and anammox bacteria.** Specific maintenance rate ($a$) was assumed to be 10% of the maximum growht rate (µ_max_) (ref. 40). $K_{S,N}$ – half saturation constant for substrate $N$; $K_{I,O_{2}}$ – inhibition constant for O_2_. Growth kinetic parameters are established assuming non-kinetic competition between metabolic activities of comammox *Nitrospira* and anammox bacteria, consistent with the values reported for anammox bacteria (18, 22).

|  | **µ_max_**  **(h^-1^)** | $\boldsymbol{a}$  **(h^-1^)** | **K_S,NH3_ (µM)** | **K_S,NO2_ (µM)** | **K_S,NO3_ (µM)** | **K_S,O2_ (µM)** | **K_I,O2_ (µM)** |
| --- | --- | --- | --- | --- | --- | --- | --- |
| AO | 0.01 | 0.001 | 1.00 | – | – | 3.13 | – |
| NO | 0.01 | 0.001 | – | 1.00 | – | 3.13 | – |
| CMX | 0.01 | 0.001 | 1.00 | – | – | 3.13 | – |
| NRMX | 0.01 | 0.001 | 1.00 | – | 1.00 | 3.13 | – |
| An-NRMX | 0.01 | 0.001 | 1.00 | 1.00 | – | – | – |
| AMX | 0.01 | 0.001 | 1.00 | 1.00 | – | – | 3.13 |
|  | | | | | | | |

**Table S5. Metabolic stoichiometries of *Nitrospira* metabolisms and anammox bacteria.**

| **Process**^(1)^ | **Compounds (M)^(2)^** | | | | | | | | | | | |
| --- | --- | --- | --- | --- | --- | --- | --- | --- | --- | --- | --- | --- |
|  | **NH_3_** | **NO_2_** | **NO_3_** | **O_2_** | **CO_2_** | **N_2_** | **AO** | **NO** | **CMX** | **NRMX** | **AnNRMX** | **AMX** |
| Growth of AO | -1/Y_X/D_ | 1/Y_X/D_ | 0 | -1.5/Y_X/D_ | -1 | 0 | 1 | 0 | 0 | 0 | 0 | 0 |
| Growth of NO | 0 | -1/Y_X/D_ | 1/Y_X/D_ | -0.5/Y_X/D_ | -1 | 0 | 0 | 1 | 0 | 0 | 0 | 0 |
| Growth of CMX | -1/Y_X/D_ | 0 | 1/Y_X/D_ | -2/Y_X/D_ | -1 | 0 | 0 | 0 | 1 | 0 | 0 | 0 |
| Growth of NRMX | -1/Y_X/D_ | 2/Y_X/D_ | -1/Y_X/D_ | -1/Y_X/D_ | -1 | 0 | 0 | 0 | 0 | 1 | 0 | 0 |
| Growth of An-NRMX | -1/Y_X/D_ | -1/Y_X/D_ | 0 | 0 | -1 | 1/Y_X/D_ | 0 | 0 | 0 | 0 | 1 | 0 |
| Growth of AMX | -1/Y_X/D_ | -1/Y_X/D_ | 0 | 0 | -1 | 1/Y_X/D_ | 0 | 0 | 0 | 0 | 0 | 1 |
| Decay of AO^(3)^ | 0 | 0 | 0 | 0 | 1 | 0 | -1 | 0 | 0 | 0 | 0 | 0 |
| Decay of NO | 0 | 0 | 0 | 0 | 1 | 0 | 0 | -1 | 0 | 0 | 0 | 0 |
| Decay of CMX | 0 | 0 | 0 | 0 | 1 | 0 | 0 | 0 | -1 | 0 | 0 | 0 |
| Decay of NRMX | 0 | 0 | 0 | 0 | 1 | 0 | 0 | 0 | 0 | -1 | 0 | 0 |
| Decay of An-NRMX | 0 | 0 | 0 | 0 | 1 | 0 | 0 | 0 | 0 | 0 | -1 | 0 |
| Decay of AMX | 0 | 0 | 0 | 0 | 1 | 0 | 0 | 0 | 0 | 0 | 0 | -1 |
| ^(1)^ Although an alternative electron donor for reduction of carbon dioxide to organic biomass precursos (anabolism) is needed, in this study the generation of oxidized electron donor from anabolism was not considered, due to the margin contribution in the nitrogen pool.  ^(2)^ Stoichiometries of substrate consumption and product synthesis are based on biomass growth yield per mole of electron donor (Y_X/D_): Y_X/NH3_ for AO, CMX, An-NRMX, AMX;Y_X/NO2_ for NO.  ^(3)^ Stochiometries for decay have been simplified at the lowest expression. | | | | | | | | | | | | |

**Table S6. Evaluation of** $\boldsymbol{\epsilon}$ **value for aerobic nitrifiers (AOB, NOB, and comammox *Nitrospira*).**

|  |  |  |  | **Calculated Y_X/eD_ (**$\boldsymbol{\epsilon}$ **= 0.258)** | |
| --- | --- | --- | --- | --- | --- |
|  | **Reported Y_X/eD_^(a)^** | **REF** | $\boldsymbol{\epsilon}$ **implied** | **Y_X/eD_^(a)^** | **Error^(b)^** |
| Ammonia-oxidizing bacteria (AOB) | | | | | |
| *Nitrosomonas europaea* | 0.0391 | (ref. 41) | 0.250 | 0.0413 | -0.06 |
| *Nitrosomonas europaea* | 0.0427 | (ref. 42) | 0.263 | 0.0413 | 0.03 |
| Nitrite-oxidizing bacteria (NOB) | | | | | |
| *Nitrobacter winogradskyi* | 0.0209 | (ref. 43) | 0.224 | 0.0262 | -0.25 |
| *Nitrobacter vulgaris* | 0.0250 | (ref. 43) | 0.251 | 0.0262 | -0.05 |
| *Nitrobacter agilis* | 0.0257 | (ref. 44) | 0.255 | 0.0262 | -0.02 |
| *Nitrobacter hamburgensis* | 0.0272 | (ref. 43) | 0.265 | 0.0262 | 0.04 |
| *Ca.* Nitrotoga arctica | 0.0257 | (ref. 43) | 0.255 | 0.0262 | -0.02 |
| *Ca.* Nitrospira defluvii | 0.0106 | (ref. 43) | 0.152 | 0.0262 | -1.45 |
| *Nitrospira marina* | 0.0243 | (ref. 45) | 0.255 | 0.0262 | -0.02 |
| *Nitrospira lenta* | 0.0303 | (ref. 43) | 0.285 | 0.0262 | 0.14 |
| *Nitrospira moscoviensis* | 0.0308 | (ref. 43) | 0.288 | 0.0262 | 0.15 |
| *Nitrospira moscoviensis* | 0.0358 | (ref. 46) | 0.320 | 0.0262 | 0.27 |
| Complete ammonia-oxidizing bacteria (comammox *Nitrospira*) | | | | | |
| *Nitrospira inopinata* | 0.0753 | (ref. 14) | 0.294 | 0.0601 | 0.20 |
| Average | – | – | 0.258 | – | – |
| Std. Dev. | – | – | 0.040 | – | – |
| Number | – | – | 13 | – | – |
| ^(a)^ Units: mol_Cx_/mol_NH3_ for AOB and CMX; mol_Cx_/mol_NO2_ for NOB.  ^(b)^ Error is calculated as: $Error= {[(Reported Y_{X/eD}) -(Predicted Y_{X/eD})]}/{(Reported Y_{X/eD})}$. | | | | | |

**Table S7. Statistical significance (*p*-value) from comparison of relative abundances of comammox *Nitrospira* metabolisms and AMX across the different oxygen concentration.** **A)** Ammonia feeding (NH_3_:NO_2_:NO_3_ = 500:0:0 µM). **B)** Equimolar feeding (NH_3_:NO_2_:NO_3_ = 500:500:500 µM). **C)** Non-equimolar feeding (NH_3_:NO_2_:NO_3_ = 500:375:500 µM). Symbol legend: ns – not significant; * – *p* < 0.05; ** – *p* < 0.01; *** – *p* < 0.001.

| **A)** | **[O_2_]** | **AO** | **NO** | **CMX** | **NRMX** | **An-NRMX** | **AMX** |
| --- | --- | --- | --- | --- | --- | --- | --- |
|  | 1.0µM / 1.5µM | ns | ** | ns | ns | ns | ** |
|  | 1.0µM / 3.0µM | ns | *** | ns | ns | *** | ** |
|  | 1.0µM / 93.8µM | ns | ** | * | ns | *** | ** |
|  | 1.5µM / 3.0µM | ns | *** | ns | ns | *** | * |
|  | 1.5µM / 93.8µM | * | *** | ** | ns | *** | * |
|  | 3.0µM / 93.8µM | * | *** | ** | ns | – | – |
|  |  |  |  |  |  |  |  |
| **B)** | **[O_2_]** | **AO** | **NO** | **CMX** | **NRMX** | **An-NRMX** | **AMX** |
|  | 1.0µM / 1.5µM | ns | * | ns | * | ns | * |
|  | 1.0µM / 3.0µM | ** | *** | *** | ns | * | *** |
|  | 1.0µM / 93.8µM | * | ** | ** | * | * | *** |
|  | 1.5µM / 3.0µM | * | ** | * | ns | * | * |
|  | 1.5µM / 93.8µM | ns | ** | ** | ns | * | * |
|  | 3.0µM / 93.8µM | ns | ns | ns | ns | – | – |
|  |  |  |  |  |  |  |  |
| **C)** | **[O_2_]** | **AO** | **NO** | **CMX** | **NRMX** | **An-NRMX** | **AMX** |
|  | 1.0µM / 1.5µM | ns | ** | * | ns | ns | ** |
|  | 1.0µM / 3.0µM | ns | ** | * | ns | * | *** |
|  | 1.0µM / 93.8µM | ns | ** | ** | ** | * | *** |
|  | 1.5µM / 3.0µM | ns | *** | * | ns | ** | *** |
|  | 1.5µM / 93.8µM | ns | *** | ** | * | ** | *** |
|  | 3.0µM / 93.8µM | ns | * | ns | ns | – | – |
|  |  |  |  |  |  |  |  |

**Table S8. Statistical significance (*p*-value) from comparison of relative abundances of comammox *Nitrospira* metabolisms and AMX across the different N feeding regimes.** **A)** Hypoxic environment with 1.0 µM O_2_. **B)** Hypoxic environment with 1.5 µM O_2_. **C)** Hypoxic environment with 3.0 µM O_2_. **D)** Aerobic enviornment with 93.8 µM O_2_. Symbol legend: ns – not significant; * – *p* < 0.05; ** – *p*< 0.01; *** – *p* < 0.001. Feeding regimes (NH_3_:NO_2_:NO_3_): ammonia feeding – 500:0:0 µM; equimolar feeding – 500:500:500 µM; non-equimolar feeding – 500:375:500 µM.

| **A)** | **Feeding regimes** | **AO** | **NO** | **CMX** | **NRMX** | **An-NRMX** | **AMX** |
| --- | --- | --- | --- | --- | --- | --- | --- |
|  | Ammonia feeding / Equimolar feeding | * | ** | * | * | ns | *** |
|  | Ammonia feeding / Non-equimolar feeding | * | ns | * | ns | ns | *** |
|  | Equimolar feeding / Non-equimolar feeding | * | ** | * | ** | ns | ns |
|  |  |  |  |  |  |  |  |
| **B)** | **Feeding regimes** | **AO** | **NO** | **CMX** | **NRMX** | **An-NRMX** | **AMX** |
|  | Ammonia feeding / Equimolar feeding | ** | ** | ** | ns | * | * |
|  | Ammonia feeding / Non-equimolar feeding | * | ** | ** | ns | * | ** |
|  | Equimolar feeding / Non-equimolar feeding | ns | * | ns | ns | ns | ns |
|  |  |  |  |  |  |  |  |
| **C)** | **Feeding regimes** | **AO** | **NO** | **CMX** | **NRMX** | **An-NRMX** | **AMX** |
|  | Ammonia feeding / Equimolar feeding | ** | *** | *** | ns | – | – |
|  | Ammonia feeding / Non-equimolar feeding | ns | ** | ns | ns | – | – |
|  | Equimolar feeding / Non-equimolar feeding | ns | ns | ns | ns | – | – |
|  |  |  |  |  |  |  |  |
| **D)** | **Feeding regimes** | **AO** | **NO** | **CMX** | **NRMX** | **An-NRMX** | **AMX** |
|  | Ammonia feeding / Equimolar feeding | ns | ** | ** | ns | – | – |
|  | Ammonia feeding / Non-equimolar feeding | ns | ** | * | ns | – | – |
|  | Equimolar feeding / Non-equimolar feeding | ns | * | ns | ns | – | – |

**Table S9. Statistical significance (*p*-value) from comparison of *Nitrospira*’s metabolism ratio represented as ln(A/B) at different nitrogen feeding regimes (NH_3_:NO_2_:NO_3_) and oxygen concentrations.** **A)** Metabolic ratios between CMX and division of labour (AO+NO). **B)** Metabolic ratios between CMX and AO. Symbol legend: ns – not significant; * – *p* < 0.05; ** – *p* < 0.01. Feeding regimes (NH_3_:NO_2_:NO_3_): ammonia feeding – 500:0:0 µM; equimolar feeding – 500:500:500 µM; Non-equimolar feeding – 500:375:500 µM.

| **A)** | **Feeding regimes** | **1.0 µM O_2_** | **1.5 µM O_2_** | **3.0 µM O_2_** | **93.8 µM O_2_** |
| --- | --- | --- | --- | --- | --- |
|  | Ammonia feeding / Equimolar feeding | ** | ns | *** | ** |
|  | Ammonia feeding / Non-equimolar feeding | ns | ns | ns | * |
|  | Equimolar feeding / Non-equimolar feeding | * | ns | ns | ns |
|  |  |  |  |  |  |
| **B)** | **Feeding regimes** | **1.0 µM O_2_** | **1.5 µM O_2_** | **3.0 µM O_2_** | **93.8 µM O_2_** |
|  | Ammonia feeding / Equimolar feeding | ns | ns | ns | ns |
|  | Ammonia feeding / Non-equimolar feeding | ns | ns | ns | ns |
|  | Equimolar feeding / Non-equimolar feeding | ns | ns | ns | ns |

**Table S10. Statistical significance (*p*-value) from comparison of relative abundances of comammox *Nitrospira* metabolisms and AMX across the different oxygen concentration.** Assuming nitrite affinity of comammox *Nitrospira* equal to **A)** 1.0 µM of NO_2_^-^, **B)** 12.5 µM of NO_2_^-^ and **C)** 449.2 µM of NO_2_^-^. Symbol legend: ns – not significant; * – *p* < 0.05; ** – *p* < 0.01; *** – *p* < 0.001.

| **A)** | **[O_2_]** | **AO** | **NO** | **CMX** | **NRMX** | **An-NRMX** | **AMX** |
| --- | --- | --- | --- | --- | --- | --- | --- |
|  | 1.0µM / 1.5µM | ns | ** | * | ns | ns | ** |
|  | 1.0µM / 3.0µM | ns | ** | * | ns | * | *** |
|  | 1.5µM / 3.0µM | ns | *** | * | ns | ** | *** |
|  |  |  |  |  |  |  |  |
| **B)** | **[O_2_]** | **AO** | **NO** | **CMX** | **NRMX** | **An-NRMX** | **AMX** |
|  | 1.0µM / 1.5µM | ns | ns | * | * | ns | * |
|  | 1.0µM / 3.0µM | * | ** | ** | ns | ns | *** |
|  | 1.5µM / 3.0µM | * | ** | ** | ns | ns | *** |
|  |  |  |  |  |  |  |  |
| **C)** | **[O_2_]** | **AO** | **NO** | **CMX** | **NRMX** | **An-NRMX** | **AMX** |
|  | 1.0µM / 1.5µM | ns | ns | ns | ns | ns | ns |
|  | 1.0µM / 3.0µM | ns | ns | ** | ns | ns | * |
|  | 1.5µM / 3.0µM | * | ns | ** | ns | ns | * |
|  |  |  |  |  |  |  |  |

**Table S11. Statistical significance (*p*-value) from comparison of relative abundances of comammox *Nitrospira* metabolisms and AMX across the different nitrite affinities of comammox *Nitrospira*.** **A)** Hypoxic environment with 1.0 µM O_2_. **B)** Hypoxic environment with 1.5 µM O_2_. **C)** Hypoxic environment with 3.0 µM O_2_. Symbol legend: ns – not significant; * – *p* < 0.05; ** – *p*< 0.01; *** – *p* < 0.001.

| **A)** | **Nitrite affinity of *Nitrospira* (K_NO2_)** | **AO** | **NO** | **CMX** | **NRMX** | **An-NRMX** | **AMX** |
| --- | --- | --- | --- | --- | --- | --- | --- |
|  | 1.0 µM NO_2_^‑^ / 12.5 µM NO_2_^‑^ | ns | ns | ns | * | * | ** |
|  | 1.0 µM NO_2_^‑^ / 449.2 µM NO_2_^‑^ | ns | ns | * | ** | * | ns |
|  | 12.5 µM NO_2_^‑^ / 449.2 µM NO_2_^‑^ | *** | ns | * | ** | ns | ** |
|  |  |  |  |  |  |  |  |
| **B)** | **Nitrite affinity of *Nitrospira* (K_NO2_)** | **AO** | **NO** | **CMX** | **NRMX** | **An-NRMX** | **AMX** |
|  | 1.0 µM NO_2_^‑^ / 12.5 µM NO_2_^‑^ | * | ** | ns | ns | ** | *** |
|  | 1.0 µM NO_2_^‑^ / 449.2 µM NO_2_^‑^ | ns | ** | ns | * | ** | *** |
|  | 12.5 µM NO_2_^‑^ / 449.2 µM NO_2_^‑^ | ns | ns | * | * | ns | ** |
|  |  |  |  |  |  |  |  |
| **C)** | **Nitrite affinity of *Nitrospira* (K_NO2_)** | **AO** | **NO** | **CMX** | **NRMX** | **An-NRMX** | **AMX** |
|  | 1.0 µM NO_2_^‑^ / 12.5 µM NO_2_^‑^ | ns | * | ns | ns | ns | ** |
|  | 1.0 µM NO_2_^‑^ / 449.2 µM NO_2_^‑^ | ns | *** | ns | ns | ns | ** |
|  | 12.5 µM NO_2_^‑^ / 449.2 µM NO_2_^‑^ | ns | *** | * | * | ns | * |
|  |  |  |  |  |  |  |  |

# References

1. McCarty PL. Thermodynamic electron equivalents model for bacterial yield prediction: modifications and comparative evaluations. Biotechnology and Bioengineering. 2007;97(2):377-88.

2. Rittmann BE, McCarty PL. Environmental biotechnology: principles and applications. Second edition. ed. New York: McGraw-Hill Education; 2020.

3. Thomas JW. Numerical partial differential equations: finite difference methods. Berlin, New York: Springer-Verlag1995. 437- p.

4. Kreft J-U, Booth G, Wimpenny JWT. BacSim, a simulator for individual-based modelling of bacterial colony growth. Microbiology. 1998;144(12):3275-87.

5. Kreft J-U, Picioreanu C, Wimpenny JWT, van Loosdrecht MCM. Individual-based modelling of biofilms. Microbiology. 2001;147(11):2897-912.

6. Briggs W, Henson V, McCormick S. A multigrid tutorial. 2nd Edition ed2000.

7. Kleerebezem R, Van Loosdrecht MCM. A generalized method for thermodynamic state analysis of environmental systems. Critical Reviews in Environmental Science and Technology. 2010;40(1):1-54.

8. Heijnen JJ, van Dijken JP. In search of a thermodynamic description of biomass yields for chemotrophic growth of microorganisms. Biotechnology and Bioengineering. 1992;39(8):833-58.

9. Samet H, editor An overview of quadtrees, octrees, and related hierarchical data structures1988; Berlin, Heidelberg: Springer Berlin Heidelberg.

10. Kreft JU, Picioreanu C, Wimpenny JWT, Van Loosdrecht MCM. Individual-based modelling of biofilms. Microbiology. 2001;147(11):2897-912.

11. Daims H, Lebedeva EV, Pjevac P, Han P, Herbold C, Albertsen M, et al. Complete nitrification by Nitrospira bacteria. Nature. 2015;528(7583):504-9.

12. van Kessel MAHJ, Speth DR, Albertsen M, Nielsen PH, Op Den Camp HJM, Kartal B, et al. Complete nitrification by a single microorganism. Nature. 2015;528(7583):555-9.

13. Martinez-Rabert E, van Amstel C, Smith C, Sloan WT, Gonzalez-Cabaleiro R. Environmental and ecological controls of the spatial distribution of microbial populations in aggregates. PLOS Computational Biology. 2022;18(12):e1010807.

14. Kits KD, Sedlacek CJ, Elena V, Han P, Bulaev A, Pjevac P, et al. Kinetic analysis of a complete nitrifier reveals an oligotrophic lifestyle. Nature Publishing Group. 2017;549(7671):269-72.

15. Sakoula D, Koch H, Frank J, Jetten MSM, van Kessel MAHJ, Lücker S. Enrichment and physiological characterization of a novel comammox Nitrospira indicates ammonium inhibition of complete nitrification. The ISME Journal. 2021;15(4):1010-24.

16. Gottshall EY, Bryson SJ, Cogert KI, Landreau M, Sedlacek CJ, Stahl DA, et al. Sustained nitrogen loss in a symbiotic association of comammox Nitrospira and anammox bacteria. Water Research. 2021;202.

17. Strous M, Kuenen JG, Jetten MSM. Key physiology of anaerobic ammonium oxidation. Applied and Environmental Microbiology. 1999;65(7):3248-50.

18. van der Star WRL, Miclea AI, van Dongen UGJM, Muyzer G, Picioreanu C, van Loosdrecht MCM. The membrane bioreactor: a novel tool to grow anammox bacteria as free cells. Biotechnology and Bioengineering. 2008;101(2):286-94.

19. Ni B-J, Chen Y-P, Liu S-Y, Fang F, Xie W-M, Yu H-Q. Modeling a granule-based anaerobic ammonium oxidizing (anammox) process. Biotechnology and Bioengineering. 2009;103(3):490-9.

20. Oshiki M, Shimokawa M, Fujii N, Satoh H, Okabe S. Physiological characteristics of the anaerobic ammonium-oxidizing bacterium 'Candidatus Brocadia sinica'. Microbiology. 2011;157(6):1706-13.

21. Puyol D, Garcia B, Field JA. Bioresource technology kinetic characterization of Brocadia spp. dominated anammox cultures. Bioresource Technology. 2013;139:94-100.

22. Straka LL. Affinity informs environmental cooperation between ammonia- oxidizing archaea (AOA) and anaerobic ammonia-oxidizing (anammox) bacteria. The ISME Journal. 2019;13(8):1997-2004.

23. Beber ME, Gollub MG, Mozaffari D, Shebek KM, Flamholz Avi I, Milo R, et al. eQuilibrator 3.0: a database solution for thermodynamic constant estimation. Nucleic Acids Research. 2022;50(D1):D603-D9.

24. Lide DR. CRC Handbook of Chemistry and Physics. 87th ed2006. 2608- p.

25. van den Berg L, van Loosdrecht MCM, de Kreuk MK. How to measure diffusion coefficients in biofilms: a critical analysis. Biotechnology and Bioengineering. 2021;118(3):1273-85.

26. Watson S. Nitrifying bacteria. Bergey's manual of systematic bacteriology. 1989;3:1808-34.

27. Loferer-Krößbacher M, Klima J, Psenner R. Determination of bacterial cell dry mass by transmission electron microscopy and densitometric image analysis. Applied and Environmental Microbiology. 1998;64(2):688-94.

28. Milo R, Philips R. Cell biology by the numbers. 1st ed. ed2015.

29. Roels JA. Energetics and kinetics in biotechnology. The Quarterly Review of Biology. 1984;59(2):179-.

30. Suarez C, Piculell M, Modin O, Langenheder S, Persson F, Hermansson M. Thickness determines microbial community structure and function in nitrifying biofilms via deterministic assembly. Scientific Reports. 2019;9(1):5110.

31. Seuntjens D, Carvajal-arroyo JM, Ruopp M, Bunse P, Mulder CPD, Lochmatter S, et al. High-resolution mapping and modeling of anammox recovery from recurrent oxygen exposure. Water Research. 2018;144:522-31.

32. Strous M, Heijnen JJ, Kuenen JG, Jetten MSM. The sequencing batch reactor as a powerful tool for the study of slowly growing anaerobic ammonium-oxidizing microorganisms. Applied Microbiology and Biotechnology. 1998;50(5):589-96.

33. Egli K, Fanger U, Alvarez PJJ, Siegrist H, van der Meer JR, Zehnder AJB. Enrichment and characterization of an anammox bacterium from a rotating biological contactor treating ammonium-rich leachate. Archives of Microbiology. 2001;175(3):198-207.

34. Oshiki M, Satoh H, Okabe S. Ecology and physiology of anaerobic ammonium oxidizing bacteria. Environmental Microbiology. 2016;18(9):2784-96.

35. Jensen M, Kuypers M, Lavik G, Thamdrup B. Rates and regulation of anaerobic ammonium oxidation and denitrification in the Black Sea. Limnology and Oceanography. 2008;53:23-36.

36. Kalvelage T, Jensen MM, Contreras S, Revsbech NP, Lam P, Günter M, et al. Oxygen sensitivity of anammox and coupled N-cycle processes in oxygen minimum zones. PLOS ONE. 2011;6(12):e29299.

37. Niederdorfer R, Fragner L, Yuan L, Hausherr D, Wei J, Magyar P, et al. Distinct growth stages controlled by the interplay of deterministic and stochastic processes in functional anammox biofilms. Water Research. 2021;200:117225.

38. Dalsgaard T, Stewart FJ, Thamdrup B, Brabandere LD, Revsbech NP, Ulloa O, et al. Oxygen at nanomolar levels reversibly suppresses process rates and gene expression in anammox and denitrification in the oxygen minimum zone of northern Chile. mBio. 2014;5(6):e01966-14.

39. Carvajal-Arroyo JM, Sun W, Sierra-Alvarez R, Field JA. Inhibition of anaerobic ammonium oxidizing (anammox) enrichment cultures by substrates, metabolites and common wastewater constituents. Chemosphere. 2013;91(1):22-7.

40. Bodegom PV. Microbial maintenance: a critical review on its quantification. Microbial Ecology. 2007;53:513-23.

41. Keen GA, Prosser JI. Steady state and transient growth of autotrophic nitrifying bacteria. Archives of Microbiology. 1987;147:73-9.

42. Martens-Habbena W, Torre RD, Stahl DA, Berube PM, Urakawa H. Ammonia oxidation kinetics determine niche separation of nitrifying archaea and bacteria. Nature. 2009;461(October).

43. Nowka B, Daims H, Spieck E. Comparison of oxidation kinetics of nitrite-oxidizing bacteria: nitrite availability as a key factor in niche differentiation. Applied and Environmental Microbiology. 2015;81(2):745-53.

44. Hunik JH, Bos CG, Hoogen MPVD, De Gooijer CD, Tramper J. Co-immobilized Nitrosomonas europaea and Nitrobacter agilis cells: validation of a dynamic model for simultaneous substrate conversion and growth in K-carrageenan gel beads. Biotechnology and Bioengineering. 1994;43:1153-63.

45. Watson SW, Book E, Valois FW, Waterbury JB, Schlosser U. Nitrospira marina gen. nov. sp. nov.: a chemolithotrophic nitrite-oxidizing bacterium. Archives of Microbiology. 1986;144(1):1-7.

46. Ehrich S, Behrens D, Lebedeva E, Ludwig W, Bock E. A new obligately chemolithoautotrophic, nitrite-oxidizing bacterium, Nitrospira moscoviensis sp. nov. and its phylogenetic relationship. Archives of Microbiology. 1995;164(1):16-23.
